# Salt Stress in *Arabidopsis*: Lipid Transfer Protein AZI1 and Its Control by Mitogen-Activated Protein Kinase MPK3

**DOI:** 10.1093/mp/sst157

**Published:** 2013-11-08

**Authors:** Andrea Pitzschke, Sneha Datta, Helene Persak

**Affiliations:** ^a^Department of Applied Genetics and Cell Biology, University of Natural Resources and Life Sciences, Muthgasse 18, 1190 Vienna, Austria

**Keywords:** salt stress, *Arabidopsis*, MAPK, MPK3, lipid transfer protein, AZI1, phosphorylation, in vivo interaction.

## Abstract

A plant’s capability to cope with environmental challenges largely relies on signal transmission through mitogen-activated protein kinase (MAPK) cascades. In *Arabidopsis thaliana*, MPK3 is particularly strongly associated with numerous abiotic and biotic stress responses. Identification of MPK3 substrates is a milestone towards improving stress resistance in plants. Here, we characterize AZI1, a lipid transfer protein (LTP)-related hybrid proline-rich protein (HyPRP), as a novel target of MPK3. AZI1 is phosphorylated by MPK3 in vitro. As documented by co-immunoprecipitation and bimolecular fluorescence complementation experiments, AZI1 interacts with MPK3 to form protein complexes *in planta*. Furthermore, null mutants of *azi1* are hypersensitive to salt stress, while *AZI1*-overexpressing lines are markedly more tolerant. *AZI1* overexpression in the *mpk3* genetic background partially alleviates the salt-hypersensitive phenotype of this mutant, but functional MPK3 appears to be required for the full extent of AZI1-conferred robustness. Notably, this robustness does not come at the expense of normal development. Immunoblot and RT–PCR data point to a role of MPK3 as positive regulator of AZI1 abundance.

## SUMMARY

Mitogen-activated protein kinase MPK3 and its novel phosphorylation target AZI1 mediate salt-stress signaling in *Arabidopsis*. AZI1 interacts with the membrane-associated pool of MPK3. MPK3 acts as positive regulator of AZI1 abundance and is required for stress tolerance conferred by *AZI1* overexpression.

## INTRODUCTION

During their lifespan, plants face a multitude of biotic and abiotic stresses. In order to survive, they need to react appropriately to modest or severe, transient or permanent, single or co-occurring stresses. In general, stresses are perceived at the cell boundary. Receptors in the plasma membrane subsequently transduce stress information to the cell body (Osakabe et al., 2013). Stress-activated transcription factors initiate a transcriptional reprogramming that finally results in changes at the protein level (Pitzschke et al., 2009b; Atkinson and Urwin, 2012; Gechev et al., 2012; Huang et al., 2012). Eventually, *de novo*-synthesized structural or enzymatically active proteins directly or indirectly pave the way to stress adaptation. Interfering with gene functions at any step in a plant’s stress signaling/adaptation response can drastically alter its stress-tolerance phenotype. Abiotic stresses, such as cold, osmotic stress, or drought, disrupt cell integrity, often accompanied by electrolyte leakage due to membrane damage, chlorophyll degradation, and accumulation of reactive oxygen species (Mahajan and Tuteja, 2005; Sinha et al., 2011; Atkinson and Urwin, 2012). To maintain cellular integrity under stress, plants have developed various adaptation strategies, including the synthesis of osmo-protectants, ‘hardening’ of the cell boundary by callose apposition, or by modifying plasma membrane composition and/or fluidity. These adaptation responses are preceded by rapidly stress-induced lipid and kinase signaling pathways (Munnik and Vermeer, 2010; Testerink and Munnik, 2011; Arisz et al., 2013).

### MAPKs and Stress Signaling

Mitogen-activated protein kinase (MAPK) cascades are conserved eukaryotic signaling modules which play key regulatory roles in development as well as in numerous stress responses. Acting early after stress perception, they serve for both amplification and transduction of the stress information. A well-studied stress-related MAPK cascade is the HOG1 pathway, which regulates the high-osmolarity response in yeast (Zi et al., 2010). Similarly, stress-related MAPK cascades have been discovered in animals (Ballif and Blenis, 2001; Kyriakis and Avruch, 2012) and plants (Rodriguez et al., 2010). Irrespective of the organism, signaling involves a phospho-relay mechanism: a MAPKKK activates its downstream MAPKK which in turn activates a target MAPK. Finally, MAPKs regulate the properties of substrate proteins through phosphorylation at serine or threonine residues adjacent to a proline (S/T-P). A kinase interaction motif (KIM; R/K-x(2–6)-I/LxI/L) found in a number of plant MAPK targets has been shown to assist substrate binding (Schweighofer et al., 2007).

Among the 10 MAPKKs and 20 MAPKs in *Arabidopsis*, MKK4/MKK5 and their direct substrates, MPK3/MPK6 in particular, are strongly associated with stress signaling. They are activated by various biotic and abiotic stimuli (Colcombet and Hirt, 2008; Pitzschke et al., 2009c; Rodriguez et al., 2010; Samajova et al., 2013). Both MKK4/MKK5 and MPK3/MPK6 are pairs of closely related proteins, which have highly, but not entirely, overlapping functions. The embryo-lethal phenotype exhibited by the *mkk4*/*mkk5* or *mpk3*/mpk6 double null mutants has largely prevented the functional characterization of these kinases (Wang et al., 2007).

Mutants such as *mkk4* that are defective in the MPK3/MPK6-upstream regulatory MAPK kinase are less tolerant to osmotic stress. In contrast, *MKK4* overexpression, which is accompanied by MPK3/MPK6 hyperactivation, enhances stress tolerance (Kim et al., 2011).

Recent studies have contributed to the understanding of active MAPK-mediated stress adaptation. In response to pathogen attack, MPK3 and MPK6 phosphorylate the transcription factor WRKY33, thereby triggering synthesis of camalexin, a major antimicrobial phytoalexin in *Arabidopsis* (Mao et al., 2011). Also, through phosphorylation of the bZIP transcription factor VIP1, MPK3 controls the expression of stress-related genes (Djamei et al., 2007), including transcription factor *MYB44* (Pitzschke et al., 2009b). The cyto-nuclear translocation of activated VIP1 appears to be a regulatory mechanism for both biotic (Djamei et al., 2007) and abiotic (Tsugama et al., 2012) stress responses. Moreover, the *MYB44* gene product itself can serve as target for MPK3 phosphorylation, suggesting a sophisticated multi-level control mechanism (Persak and Pitzschke, 2013). *MYB44* overexpression confers abiotic stress tolerance in a phosphorylation-dependent manner, but, unlike VIP1, MYB44 is always found in the nucleus, irrespective of its phosphorylation status (Persak and Pitzschke, 2013). MAPKs may further regulate plant cell shapes by interacting with or regulating cortical microtubules, as was shown for MPK4 (Beck et al.,2010, 2011), MPK6 (Muller et al., 2010), and MPK12/MPK18 (Walia et al., 2009).

### Lipid Transfer Proteins

Plant lipid transfer proteins (LTPs) are small (7–9kDa), proteins capable of exchanging lipids between membranes in vitro. However, there is still no generalized theory as to their in vivo function (Yeats and Rose, 2008). Plant LTPs are evolutionarily distinct from animal LTPs and, along with thionins and snakins, represent a plant-specific class within the group of small cysteine-rich peptides (Silverstein et al., 2007). Each class has a characteristic number and arrangement of cysteine residues. LTPs are common to flowering plants where they have been implicated in a variety of processes, including direct antimicrobial defense (Segura et al., 1993; Molina and Garcia-Olmedo, 1997; Regente et al., 2005), defensive signaling (Buhot et al., 2001; Maldonado et al., 2002; Jung et al., 2009; Yu et al., 2013), cuticle synthesis (Hollenbach et al., 1997; Cameron et al., 2006), cell-wall loosening (Nieuwland et al., 2005), and pollen tube growth (Park and Lord, 2003). The LTP gene family in *Arabidopsis* has 276 members (Silverstein et al., 2007), many of which are expressed in a tissue-, age-, and/or stimulus-specific manner (Jose-Estanyol et al., 2004; Zimmermann et al., 2004).

### EARLI-Type Hybrid Proline-Rich Proteins

Hybrid proline-rich proteins (HyPRPs) are putative cell-wall proteins characterized by the presence of a variable N-terminal domain and a conserved C-terminal domain that is related to so-called non-specific LTPs (ns-LTPs) (Dvorakova et al., 2012). Ns-LTPs bind and catalyze transfer of diverse lipids in vitro, but their in vivo function is unknown (Lindorff-Larsen et al., 2001). Compared to classical LTPs (see above), HyPRPs carry an additional proline-rich domain after the signal peptide (Jose-Estanyol et al., 2004; Silverstein et al., 2007). The *Arabidopsis* and rice genomes encode 29 and 31 HyPRPs, respectively, which exhibit signs of recent diversifications involving several independent tandem gene duplications (Dvorakova et al., 2012). In *Arabidopsis*, four highly homologous members, named EARLI-type HyPRPs are involved in the response to cold stress (Zhang and Schlappi, 2007). EARLI-type HyPRPs (At4g12470–12500) form a cluster on chromosome 4, and their expression is up-regulated by exposure to low temperatures. RNAi lines suppressed in *EARLI* (At4g12480) expression are less tolerant to freezing stress (Zhang and Schlappi, 2007). Furthermore, *EARLI1* was implicated in the osmotic stress response because *earli1*-null mutants are hypersensitive to NaCl treatment, while tolerance is enhanced upon *EARLI1* overexpression (Xu et al., 2011a). Similarly, overexpression of *AZI1* (At4g12470) was shown to improve *Arabidopsis* freezing tolerance (Xu et al., 2011b). Possible roles of *AZI1* in other abiotic stresses are as-yet unknown.

EARLI-type HyPRPs have a bimodular structure—characterized by a proline-rich domain (PRD) and an eight-cysteine motif (8CM). While the hydrophilic PRD likely confers cell-wall binding, the highly lipophilic 8CM domain is thought to interact with the plasma membrane (Zhang and Schlappi, 2007). EARLIs form higher-order complexes in plants. In SDS gels, even under highly reducing conditions, these proteins were found to migrate at a significantly larger than the expected size. β-mercapto-resistant disulfide bridges are suspected to contribute to the protein’s gel migration characteristics (Zhang and Schlappi, 2007). EARLIs may undergo additional posttranslational modifications. In fact, several putative phosphorylation sites are contained in EARLI1 protein sequences (Zhang and Schlappi, 2007). It is currently unknown whether and by which kinase EARLIs might be phosphorylated. Nor is it known how phosphorylation may affect EARLI protein function.

Through a combined survey of literature and bioinformatic data, we hypothesized EARLIs, in particular AZI1, to act directly downstream of MPK3 in stress responses. (1) *AZI1* gene expression and MPK3 activity profile overlap: a diversity of stresses induces *EARLI* expression (GENEVESTIGATOR) (Zimmermann et al., 2004); likewise, *MPK3* transcript, MPK3 protein levels, and activity increase in a stress-dependent manner (Colcombet and Hirt, 2008; Pitzschke et al., 2009c; Rodriguez et al., 2010; Samajova et al., 2013). (2) Gel migration properties of EARLI proteins are indicative of posttranslational modification(s) (Zhang and Schlappi, 2007). (3) EARLI primary protein structures contain putative sites for MAPK binding and phosphorylation. (4) The plant phosphorylation site database, PhosPhAt (Zulawski et al., 2013), predicts a site of clustered phosphorylation events, a so-called ‘phosphorylation hot spot’ in the AZI1 sequence. This hot spot comprises putative MAPK-phosphorylation motifs. (5) *azi1* mutants are specifically compromised in systemic immunity triggered by pathogens (Jung et al., 2009)—a process for which also MPK3 is indispensable (Beckers et al., 2009).

Here, we report the HyPRP AZI1 to be a novel direct target of MPK3 in *Arabidopsis* salt-stress response. AZI1 is phosphorylated by MPK3 in vitro, and it interacts with MPK3 to form protein complexes *in planta. AZI1* overexpression in *Arabidopsis* strongly improves tolerance to high-salinity conditions. It partially alleviates the salt-hypersensitive phenotype in *mpk3* mutants. Notably, AZI1-conferred resistance does not come at the expense of normal development. Our data point to a role of MPK3 as positive regulator of AZI1 abundance.

## RESULTS

**Figure 1.**
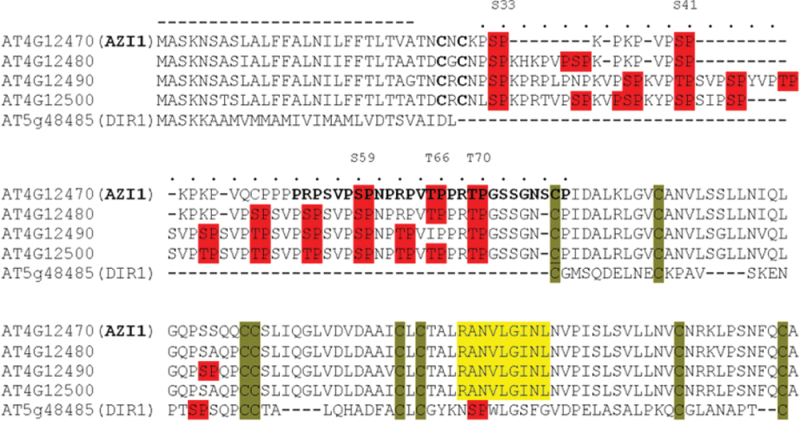
Protein Sequence Alignment of AZI1, Its Most Closely Related HyPRPs, and the Distantly Related LTP DIR1. The cysteine residues in the 8-cysteine-signature characteristic for LTPs and HyPRPs are highlighted (green). Dipeptide motifs presenting potential MAPK-phosphorylation sites (Ser-Pro, Thr-Pro) are highlighted in red. Putative MAPK interaction sites matching the consensus (R/K x_2–6_ L/IxL/I) are shown in yellow. A region in the AZI1 protein sequence, predicted as ‘phosphorylation hot spot’ (PhosPhAt, 2013), is shown in bold. The putative secretion signal and proline-rich domain are indicated by a dashed or dotted line, respectively.

A minimal criterion for a candidate MAPK substrate protein is the presence of Ser-Pro or Thr-Pro motif(s). In addition, peptides matching the KIM (R/K-X(2–6)-I/L-X-I/L; Tanoue and Nishida, 2003) are further indicators, although not a strict requirement of MAPK binding. Putative KIMs as well as multiple Ser-Pro and Thr-Pro dipeptides are contained in the four EARLI protein sequences. Whereas the majority of LTPs carry several Ser-Pro or Thr-Pro sites, KIMs are much less abundant. Interestingly, most HyPRPs but only a few non-HyPRP-type LTPs contain putative KIM(s). The sequence alignment (Figure 1) shows the four members of the EARLI HyPRP family as well as the distantly related DIR1, a well-characterized LTP involved in the pathogen response (Maldonado et al., 2002).

To investigate a possible connection between EARLI-dependent abiotic stress tolerance and MPK3-mediated stress signaling, we selected AZI1 (At4g12470), the shortest member of the EARLI cluster. *AZI1* (but none of the other *EARLI* family members) as well as *MPK3* belong to the so-called ‘multiple stress-responsive genes’ (MSTs) that had been identified through a functional-genomics-based screen of *Arabidopsis* abiotic stress responses (Kant et al., 2008). The list of MSTs responding to at least six types of abiotic stress contains 13 members of the LTP family (Supplemental Table 1). Interestingly, five of these, including AZI1, belong to the only subfamily of cysteine-rich proteins (CRP4820; 31 members) for which a striking conservation between monocots (rice) and dicots (*Arabidopsis*) has been noted (Silverstein et al., 2007).

### AZI1 Is a Binding Partner and Substrate of MPK3

**Figure 2.**
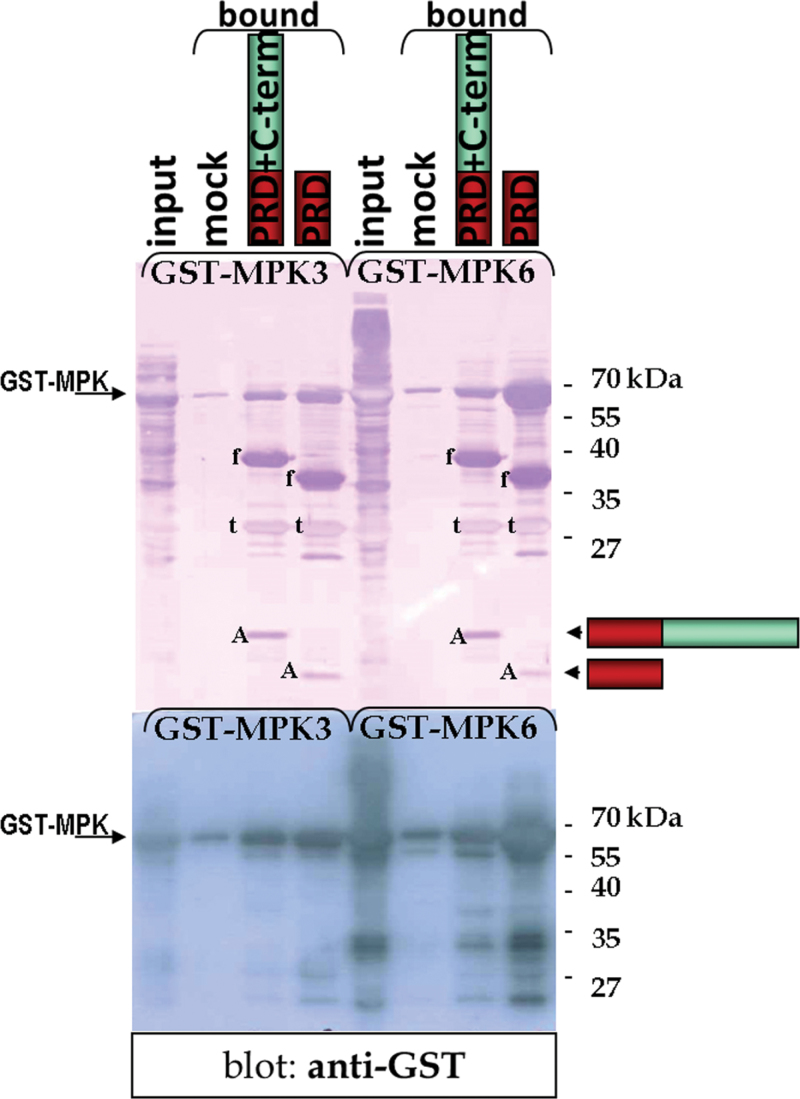
Binding of MPK3 and MPK6 to Immobilized AZI1—Pull-Down Assay. Lysates of *E. coli* expressing GST–MPK3 or GST–MPK6 (input) were incubated with chitin agarose without (‘mock’) or with AZI1 peptides immobilized by a chitin-binding-protein tag. Captured proteins were separated by SDS–PAGE and visualized by Coomassie Blue staining (top) or analyzed by immunoblotting with anti-GST antibody (bottom). The protein bands (‘f’) derive from AZI1 *f*usion proteins, which are partially cleaved into the intein–chitin-binding-protein tag (‘t’) and non-tagged AZI1 peptides (‘A’).

The hypothesized interaction between MPK3 and AZI1 was examined by in vitro pull-down assays. To this end, recombinant fusion proteins of glutathione S-transferase (GST) to MPK3 or intein–chitin-binding-protein to AZI1 were expressed in *Escherichia coli*. Attempts to produce full-length AZI1 protein failed, which might be attributable to the antimicrobial activities ascribed to several LTPs (Carvalho and Gomes, 2007). However, proteins were produced successfully when the putative secretion signal was omitted. Also, a peptide comprising the PRD only was expressed efficiently. AZI1 peptides were immobilized on chitin agarose, which was subsequently incubated with cell lysates from GST–MPK3-expressing *E. coli*. The MAPK was successfully captured by immobilized truncated AZI1 proteins (Figure 2), demonstrating physical association of MPK3 with AZI1 in vitro. Interestingly, under these conditions, the PRD of AZI1 (lacking the putative KIM highlighted in Figure 1) is sufficient for the interaction. Given the known sequence similarity and overlapping functionality between MPK3 and MPK6, pull-down experiments were also performed with GST-tagged MPK6 (Figure 2, right). Although MPK6 displayed a similar AZI1-interacting ability to MPK3, we continued to focus on MPK3.

**Figure 3.**
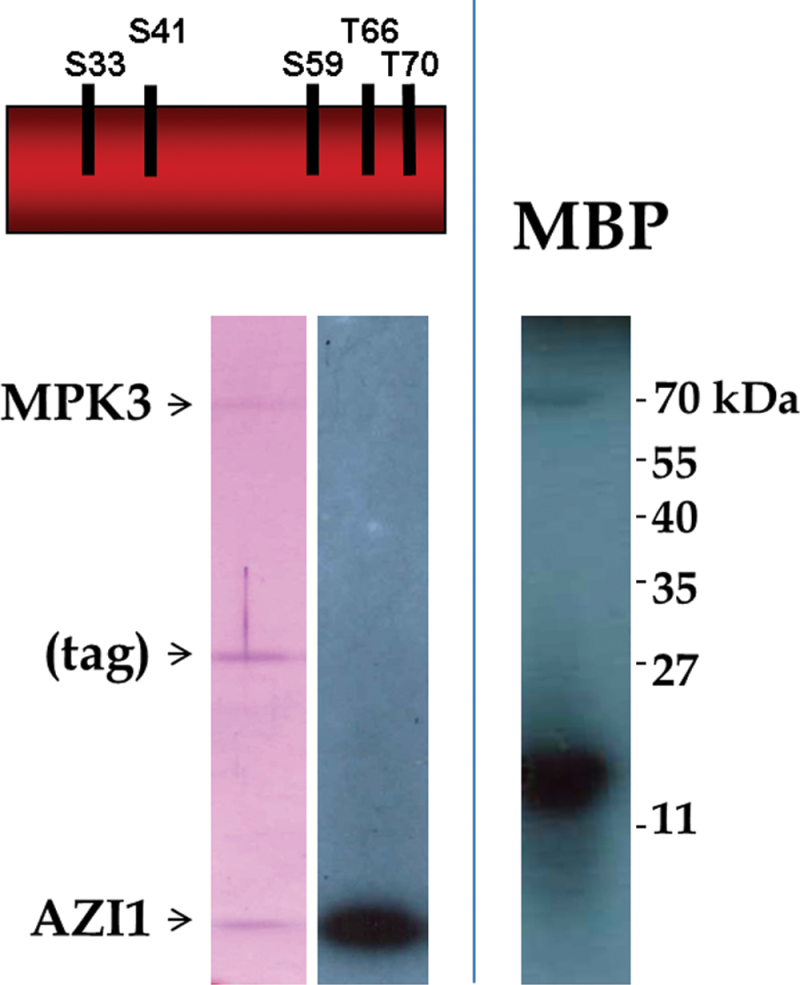
MPK3 Phosphorylates AZI1—In Vitro Kinase Assay. Recombinant proteins of AZI1 (proline-rich domain; the five putative phosphorylation sites are indicated) and GST–MPK3 were isolated from *E. coli* and incubated in kinase reaction buffer containing [γ-^32^P]-ATP. Myelin basic protein (MBP), a known MAPK substrate, served as positive control. SDS–PAGE followed by autoradiography revealed incorporation of ^32^P into AZI1 peptide and MBP (right). Left: Coomassie Blue staining of the corresponding gel section. (During protein purification, the fusion tag (intein–chitin binding domain) partially co-elutes with AZI1 peptides.)

To assess whether AZI1 is not only a binding partner, but also a substrate of MPK3, in vitro kinase assays were conducted. The intein cleavage strategy (NEB) was employed to produce tag-free AZI1 peptide. MPK3 was produced as GST–MPK3, a fusion protein with well-documented kinase activity (Djamei et al., 2007; Persak and Pitzschke, 2013). Myelin basic protein, a common MAPK substrate, was included to document functionality of the kinase. Purified recombinant proteins were incubated in the presence of gamma ^32^P-labelled ATP and subsequently separated by SDS–PAGE. Autoradiography revealed incorporation of the radioisotope into AZI1 peptide, demonstrating its phosphorylation by MPK3 (Figure 3).

### MPK3 Subcellular Localization and MPK3/AZI1 Interaction In Vivo

**Figure 4.**
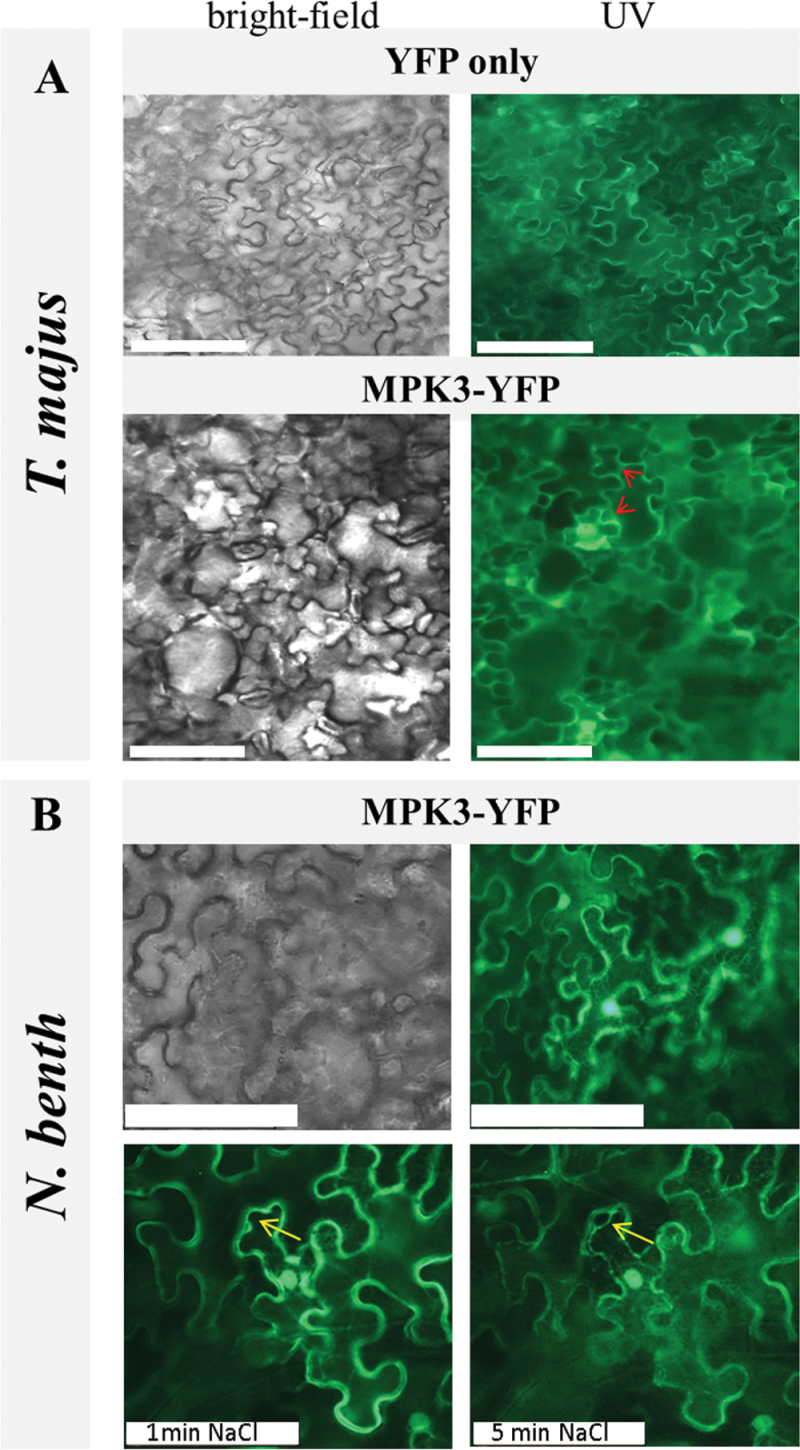
Subcellular Localization of MPK3 *In Planta*. Transient expression of free YFP (top) or MPK3–YFP (bottom) in agro-infiltrated *T. majus* **(A)**. Regions of putative MPK3 membrane associations are indicated by arrows. **(B)** Transient expression of MPK3–YFP and plasmolysis studies in *N. benthamiana*. *MPK3–YFP* localization was documented by UV microscopy 4 d post infiltration directly (top, right) and 1 or 5min after treatment of leaf discs with a 2-M NaCl solution (bottom). The progressive detachment of the plasma membrane in NaCl-treated cells is marked by an arrow. Scale bar: 100 μm.

The results from pull-down and in vitro kinase assays prompted us to investigate whether MPK3 and AZI1 interacted in vivo. The minimum requirement for such bipartite complex formation would be a certain overlap of the two protein’s subcellular location. EARLI-type HyPRPs have a predicted membrane location (SUBA; Heazlewood et al., 2007), which is in line with the reported membrane-association of EARLI1–yellow fluorescent protein (YFP) fusion proteins (Zhang and Schlappi, 2007). Similarly, AZI1 was found in the membrane-enriched pool of proteins identified by a proteomic approach (Fernandez-Calvino et al., 2011). In addition, a very recent study employing *Agrobacterium*-mediated transformation of *Nicotiana benthamiana* located AZI1–GFP fusion proteins in the endoplasmic reticulum and in plasmodesmata (Yu et al., 2013). So far, none of these sites has been directly linked to MPK3. To investigate a possible co-localization of MPK3 with membrane-bound protein, a MPK3 fusion to the YFP was expressed *in planta*, using *Agrobacterium*-mediated transformation of leaves. Free YFP or an empty vector construct served as positive and negative control, respectively. Localization studies were conducted in *Nicotiana benthamiana* and our recently developed expression system, agroinfiltration of *Tropaeolum majus* (Pitzschke, 2013). *T. majus* yields high transformation efficiencies and—being a member of the order Brassicales—is more closely related to *Arabidopsis* than is *Nicotiana* (Pitzschke, 2013). Both in *T. majus* and *N. benthamiana*, MPK3 was detected in cytoplasm and nuclei (Figure 4), which is in line with earlier reports (Brock et al., 2010; Persak and Pitzschke, 2013). In addition, a small proportion of MPK3–YFP appeared to be membrane-associated, suggesting that there is more to MPK3 localization than the reported (predominant) cyto-nuclear distribution. Plasmolysis experiments in MPK3–YFP-expressing tissue corroborate this finding (Figure 4B, bottom). These were conducted in *Nicotiana* only, because the high water repellence (‘Lotus effect’) of *T. majus* leaves would hamper treatment with liquids (Pitzschke, 2013). *Nicotiana* epidermal cells have a reportedly large vacuole which presses all other organelles against the rigid cell wall (Lopez-Marques et al., 2012). Under hyperosmotic conditions, the tonoplast freely shrinks, while, at certain contact points, the plasma membrane remains attached to the cell wall (Oparka, 1994). In NaCl-treated cells MPK3–YFP fluorescence was observed along the contracting plasma membrane, which showed the typical pattern of detachment areas and cell-wall contact points known from other studies on plasma membrane-located proteins (Lopez-Marques et al., 2012).

In summary, subcellular distribution of MPK3 partially overlaps with the reported plasma membrane-association of AZI1, thus meeting the minimal requirement of a physical MPK3-AZI1 association *in planta*.

**Figure 5.**
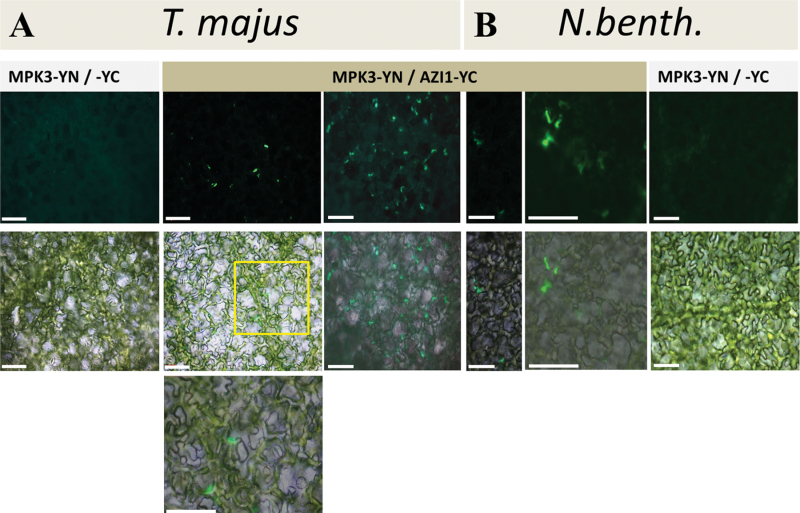
AZI1–MPK3 Interaction *In Planta*. *T. majus* **(A)** or *N. benthamiana* **(B)** leaves were infiltrated with agrobacteria carrying constructs for the constitutive expression of AZI1 and MPK3, fused to the C- and N-terminal fragment of YFP, respectively. Bimolecular fluorescence complementation was detected by UV microscopy 5 dpi; top: UV image, middle: UV/brightfield image overlay. The indicated area was enlarged and is displayed in the bottom image. No signal was obtained when either of the partners was co-infiltrated with the respective empty vector control (exemplarily shown for AZI1-YC + free YN). Scale bar = 100 μm.

Agroinfiltration of *N. benthamiana* and *T. majus* was subsequently employed to investigate MPK3/AZI1 interaction by the bimolecular fluorescence complementation (BiFC) approach. Constructs for the constitutive expression of AZI1 and MPK3, fused to the C- and N-terminal fragment of YFP, respectively, were co-delivered into leaves. Five d post infiltration, the leaf tissue was examined by UV microscopy. No signal was detectable in control co-infiltrations (AZI1–cYFP/nYFP empty vector or MPK3–nYFP/cYFP empty vector). In contrast, AZI1–cYFP/MPK3–nYFP-co-infiltrated tissue gave rise to positive BiFC formation, in *T. majus* (Figure 5A) as well as in *N. benthamiana* (Figure 5B), indicating physical association of AZI1 with the kinase *in planta.* In both species, complemented fluorescence was confined to distinct regions along the cell boundary (see the ‘Discussion’ section).

**Figure 6.**
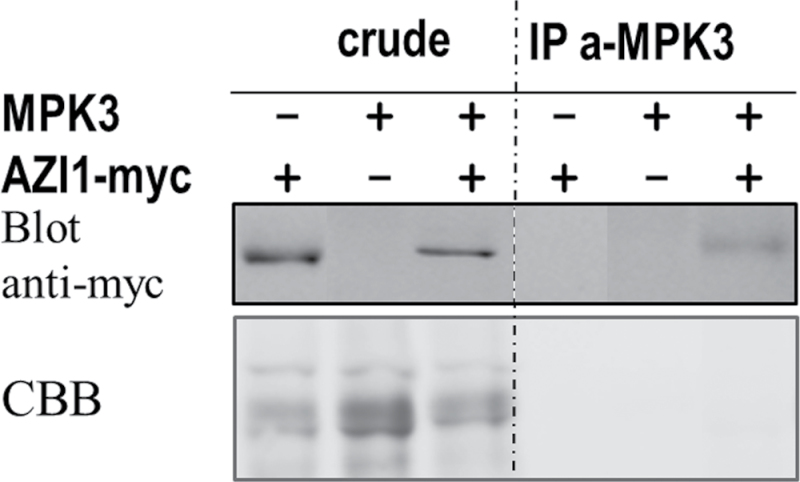
AZI1 Co-Immunoprecipitates with MPK3. MPK3 alone or in combination with AZI1–myc was transiently expressed in *N. benthamiana* leaves. Five days post transformation, proteins were extracted and incubated with anti-MPK3 antibody. Captured proteins were analyzed by immunoblot using anti-myc antibody. Protein loading was visualized by Coomassie Blue staining (CBB) of the membranes.

The AZI1/MPK3 in vivo interaction was further substantiated by co-immunoprecipitation experiments. To this end, constructs for constitutive expression of AZI1–myc and MPK3 were co-delivered into *N. benthamiana* leaves by agroinfiltration. (Co-immunoprecipitation experiments in *T. majus* leaves failed so far. The experimental parameters (buffer composition, incubation times, etc.) remain to be established in this novel expression system.) Proteins were isolated from *N. benthamiana* leaves 5 d post infiltration. AZI1–myc protein was detected in crude protein extracts and also in samples after immunoprecipitation with anti-MPK3 antibody (Figure 6B), corroborating the microscopy results (Figure 5).

Together, BiFC and co-immunoprecipitation experiments support the in vitro data and suggest a new site of action for MPK3. To learn more about the role of AZI1 and the functional relevance of AZI1/MPK3 interaction, transgenic *Arabidopsis* lines were generated and analyzed as outlined in the following paragraphs.

### *AZI1* Overexpression Enhances Salt-Stress Tolerance

**Figure 7.**
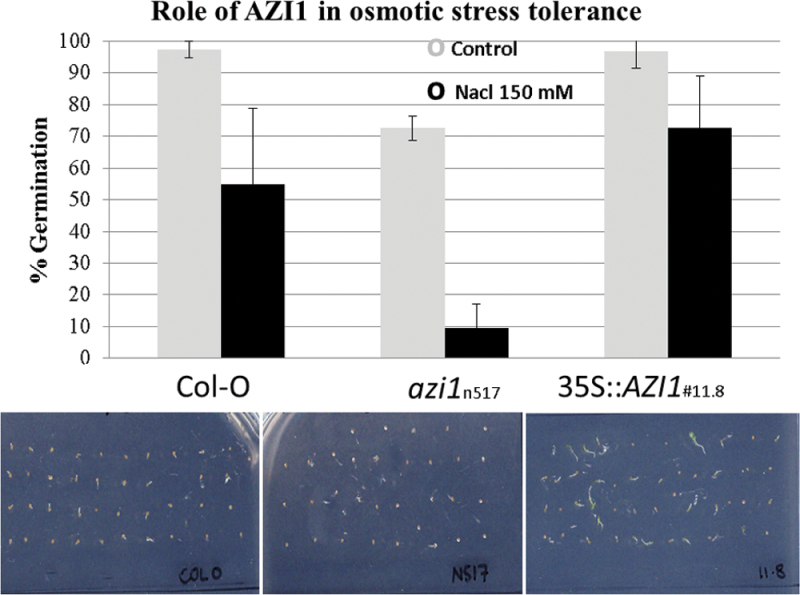
Seed Germination under High-Salinity Conditions. Seeds of wild-type, *azi1* mutant, or AZI1-overexpressing lines were germinated on standard medium in the absence or presence of 150mM NaCl. Tolerance was assessed 3 d later as percentage of germinated seeds (radicle protrusion). Average and standard deviation was calculated from three separate experiments performed with 40–50 seeds per line. Bottom panel: plate scan of one representative experiment (day 3, 150mM NaCl). Note that, although emerged radicles are seen in Col-O control and 35S::*AZI1*, the latter grows more vigorously.

So far, a role of EARLIs in plants exposed to osmotic stress has only been shown for *EARLI1* (At4g12480) (Zhang and Schlappi, 2007). The documented salt-hypersensitive phenotypes of *mpk3* (Persak and Pitzschke, 2013) and *mkk4* mutants impaired in MPK3 activation (Kim et al., 2011), as well as the observed AZI1 phosphorylation by MPK3 (Figure 3), prompted us to study salt-stress tolerance in plants lacking or overexpressing *AZI1*. Transgenic *Arabidopsis* plants ectopically expressing *AZI1*–myc fusions from the *CaMV*35S promoter were generated. Positive transformants were identified by immunoblotting and propagated to homozygosity. Homozygous *azi1* mutant lines (SALK_017709; accession number N517709), which carry a T-DNA insertion in the 3′ UTR and lack any detectable *AZI1* transcript (Xu et al., 2011b), were identified by genotyping. RT–PCR analysis verified the lack of transcript in this mutant line (Supplemental Figure 1). To get a first insight into a possible role of AZI1 in salt response, seeds were plated on medium supplemented with or without 150mM NaCl (Figure 7). With the exception of *azi1*, which had an overall reduced germination frequency, seeds germinated well on standard (half-strength MS) medium. Under salt-stress conditions, *AZI1*-overexpressing seeds germinated better than Col-0 wild-type controls. In contrast, *azi1* seeds were hypersensitive to the treatment, even after normalizing for the overall reduced germination frequency on control medium. Growth in *azi1* seedlings that did germinate on NaCl appeared to be entirely blocked, while Col-O and *35S::AZI1* formed a short root. These observations indicate that ectopic *AZI1* expression improves germination under high-salinity conditions, and that *AZI1 is* required for germination and subsequent development under these conditions.

### *AZI1* Overexpression Partially Improves Salt-Stress Tolerance in *mpk3*

#### Germination on NaCl

**Figure 8.**
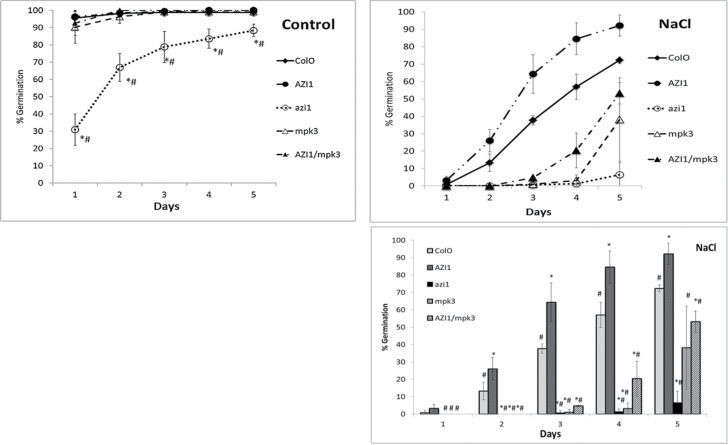
AZI1 Improves Germination under High-Salinity Conditions. Seeds (*n* = 50) of Col-O, 35S::*AZI1*, *azi1*, *mpk3*, and 35S::*AZI1/mpk3* were germinated standard medium in the absence or presence of 150mM NaCl. For purposes of clarity, the NaCl stress data are shown both as line graph and as bar graph. Tolerance was assessed as percentage germinated seeds (radicle emergence) at various time points ranging from day 1 to day 5. Average values from three independent sublines of each genotype were used. The experiment was repeated three times. *t*-test for significant difference (at *p* ≤ 0.05) of means between Col-O and individual line (*) and between 35S::*AZI1* and individual line (#) were calculated for each time point (Supplemental Table 2). Bars represent ±SD.

Our in vitro (Figures 2 and 3) and in vivo (Figures 5 and 6) protein–protein interaction data as well as the marked positive effect of *AZI1* overexpression on stress tolerance (Figure 7) raised the question: Does AZI1 improve stress tolerance in a MPK3-dependent manner? To address this, a homozygous line (named ‘11.8’) strongly expressing the *AZI1* transgene was crossed with *mpk3* mutant plants. Individuals carrying both the *MPK3*-disrupting T-DNA insertion and the *AZI1* transgene (35S::*AZI1*/*mpk3*) were identified, propagated to homozygosity, and confirmed by genotyping. Seeds were subsequently subjected to germination assays as described above (three independent repeats, using three or four sublines per genotype). *AZI1* overexpression significantly enhanced germination on NaCl over all the other lines for all the time points (except for Col-O on day 1) which was supported by results of the *t*-test (*p* ≤ 0.05) between *AZI1* and individual lines. Col-O performed significantly better (*p* ≤ 0.05) than *mpk3*, *azi1*, and *AZI1/mpk3*. Overexpression of *AZI1* in the *mpk3* background slightly improved its germination, as it showed borderline significance only at the day 4 (*p* = 0.058) time point. Thus, a functional MPK3 seems to be important for acquiring the full *AZI1* overexpression-related improved salt-stress tolerance (Figure 8).

#### Seedling Survival under High-Salinity Conditions

**Figure 9.**
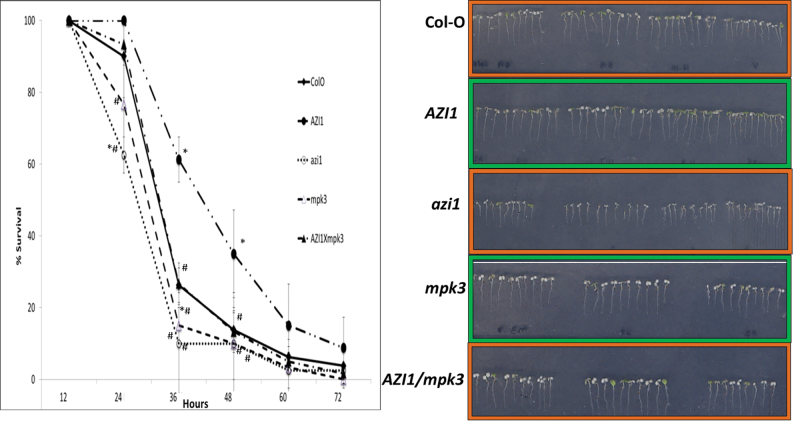
Seedling Survival under High-Salinity Conditions. Survival graph for 3-day-old seedlings of Col-O, 35S::*AZI1*, *azi1*, *mpk3*, and 35S::*AZI1/mpk3* germinated on standard half-strength MS + 0.25% sucrose and transferred onto medium supplemented with 250mM NaCl. Survival was assed as percentage survival (green cotyledons) at various time points ranging from 12h to 72h. Average values from three independent sublines of each genotype were used. The experiment was repeated three times. *t*-test for significant difference (at *p* ≤ 0.05) of means between Col-O and individual line (*) and between 35S::*AZI1* and individual line (#) were calculated for each time point (Supplemental Table 2). Bars represent ±SD. Right: photograph taken at 36-h time point.

As described above, the individual lines responded differently to high-salinity conditions. However, poorer germination does not necessarily mean that a plant is generally more salt-sensitive. Examples exist where better germination under salt stress does not correlate well with salinity tolerance at later developmental stages (reviewed by Verslues et al. (2006)). To address the aspect of salt tolerance by an alternative approach, seedlings were germinated in the absence of stress and subsequently transferred as 3-day-old seedlings onto medium supplemented with 250mM NaCl (Figure 9). Consistently with recent data (Persak and Pitzschke, 2013), *mpk3* mutants displayed a salt-hypersensitive phenotype. Survival was best for 35S::*AZI1* lines, and lowest for *azi1* and *mpk3* mutant lines. *AZI1* overexpression significantly enhanced survival under high-salinity conditions over all the other lines, especially for the 36-h and 48-h time points, which was supported by results of the *t*-test (*p* ≤ 0.05) between *AZI1* and individual lines (Figure 9 and Supplemental Table 2). 35S::*AZI1/mpk3* had tolerance levels comparable to Col-O. Thus, overexpression of *AZI1* improved salt tolerance in *mpk3*, but it was not statistically significant. 35S::*AZI1/mpk3* seedlings did not reach tolerance levels of the *35S::AZI1* line. Thus, a functional MPK3 seems to be required to reach the full extent of *AZI1*-conferred salt-stress tolerance. A role of *AZI1* acting downstream of MPK3 in salt-stress signaling may therefore be considered. In this scenario, one or more properties of the AZI1 protein are likely to differ, depending on the genetic background (*MPK3* ^*+/+*^ or *mpk3* ^*–/–*^) in which the transgene is expressed.

### MPK3 Controls AZI1 Protein Abundance

**Figure 10.**
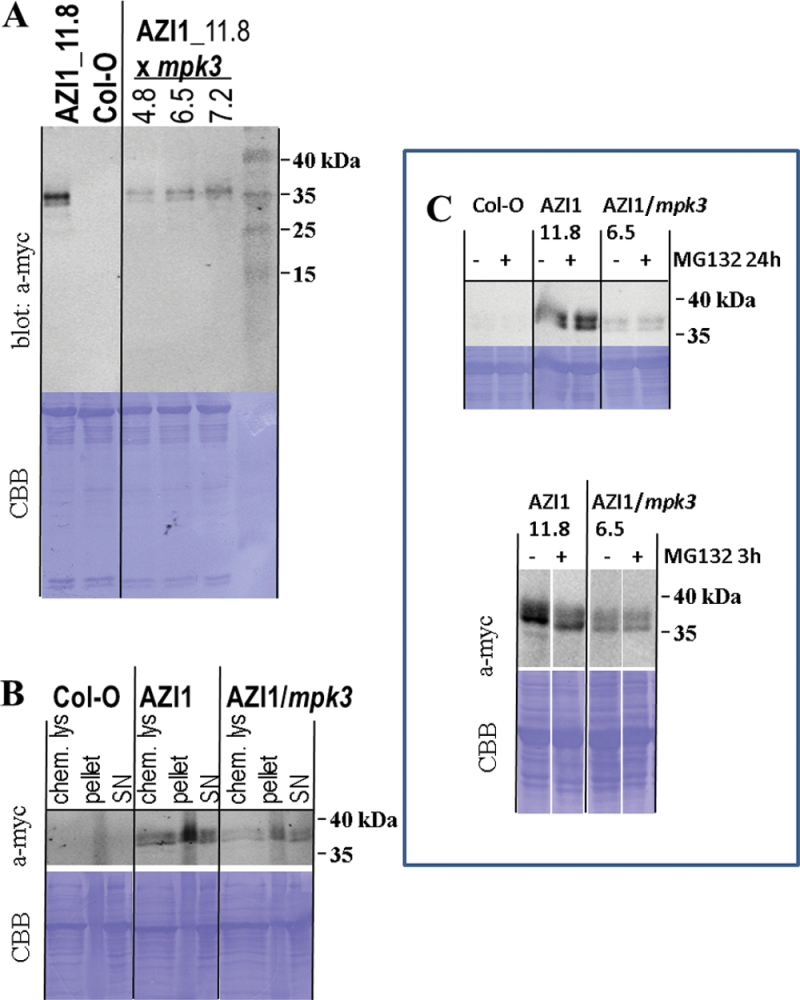
Immunoblot Analyses in Transgenic *Arabidopsis*. **(A)** AZI1–myc protein levels are reduced in *mpk3* mutants. Proteins were extracted from seedlings of 35S::*AZI1* and from 35S::AZI1/*mpk3* (homozygous crossings of line 35S::*AZI1*_11.8 with *mpk3*). AZI1–myc expression was detected by immunoblotting with an antibody directed against the myc epitope tag. Protein loading was visualized by subsequent Coomassie Blue staining of the membrane (CBB). Homozygous 35S::AZI1/*mpk3* lines (three are shown) have consistently less AZI1–myc protein. The experiment was repeated three times, with similar results. **(B)** Ectopically expressed AZI1 protein has similar solubility in Col-O and *mpk*3. Proteins of Col-O, 35S::AZI1, and 35S::AZI/*mpk3* 14-day-old seedlings were extracted by chemical lysis or by a ‘classic’ procedure (see the ‘Methods’ section). Pellets and supernatent fluid (SN) were loaded separately. AZI1 was detected by immunoblotting with anti-myc antibody. The additional ‘smear’ flanking the 36/38-kDa region in the three ‘pellet’ lanes likely derives from non-specific immunoreactive compounds. Two independent repeats of the experiment yielded similar results. **(C)** AZI1 protein does not accumulate upon inhibition of the 26S proteasome degradation machinery. Seedlings of Col-0, *35S::AZI1 and 35S::AZI1/mpk3* were treated with 50 μM MG132 for 24h (top) or 3h (bottom). AZI1–myc protein was detected by immunoblot analysis as described in (A).

To study AZI1 protein properties potentially regulated by MPK3, protein extracts of 35S::*AZI1* and 35S::*AZI1*/*mpk3* seedlings were analyzed by immunoblotting using antibodies against the myc epitope tag (Figure 10A). Qualitatively, there was no discernible difference between samples from 35S::*AZI1* and 35S::*AZI1*/*mpk3* lines. In both genotypes, AZI1–myc protein migrated as 36kDa/38kDa double band, and thus several kDa higher than the expected size. Depending on cleavage of the N-terminal secretion peptide, the calculated size (including the 14-kDa myc-tag) is 31.9kDa or 29.4kDa, respectively. This marked differences in expected versus actual size are indicative of posttranslational modification(s) that are eukaryotic-specific, because recombinant AZI1 proteins produced in *E. coli* (Figure 2) migrated normally. This issue was addressed by *in silico* analysis (supplementary data) as part of the discussion section. Interestingly, compared to samples from 35S::*AZI1* plants, signal intensity was consistently lower in each of the three independent 35S::*AZI1*/*mpk3* lines that were tested. This effect was apparently independent of plant age or growth conditions, since it was observed in protein extracts of variously aged (5-, 7-, and 14-day-old) aseptically grown seedlings as well as of soil-grown adult plants (not shown).

Compared to the parental 35S::*AZI1* line, AZI1-overexpressing lines that had been obtained (along with the 35S::*AZI1/mpk3* ^*−/−*^ lines) from crossings but that were genotyped as homozygous 35S::*AZI1/MPK3* ^*+/+*^ contained similar amounts of the AZI1–myc protein (Supplemental Figure 2). They also showed improved survival on NaCl, similar to the parent line *35S::AZI1.* Thus, reduced AZI1–myc levels in 35S::*AZI1/mpk3* correlate with *MPK3* deficiency; they unlikely derive from non-specific loss of expression that might have occurred during seed propagation.

If MPK3 had a fundamental influence on AZI1 protein properties, the different signal intensities seen on immunoblots might be due to altered accessibility to the extraction procedure. To test this, pellets and supernatant fluid of protein extracts were examined in parallel (see the ‘Methods’ section). In addition, aliquots of seedlings were processed for protein extraction by the ‘chemical lysis method’ (where entire seedlings are directly boiled in extraction buffer) (Tsugama et al., 2011). The relative distribution of the AZI1–myc protein between pellets versus supernatant fluid was similar in 35S::*AZI1* and 35S::*AZI1*/*mpk3* (Figure 10B); a major proportion of the protein was found in the insoluble fraction. AZI1–myc protein levels in the three sample types (supernatant fluid, pellet, or chemical lysate) were consistently higher in 35S::*AZI1*/MPK3 as compared to 35S::*AZI1*/*mpk3*.

To investigate the possibility that the differences in AZI1–myc protein abundance were due to an overall impairment of protein biosynthesis and integrity in *mpk3*, the general protein profiles of all transgenic and mutant lines described above were compared. Protein extracts of duplicate samples were separated by SDS–PAGE. As revealed by Coomassie Blue staining (Supplemental Figure 3), there was no discernible difference between the samples tested, suggesting that manipulation of MPK3, AZI1, or both has no major impact on protein synthesis or stability. Conclusively, recombinant AZI1 protein appeared to be less abundant in the *mpk3* mutant than in the *MPK3* wild-type background, while its gel migration properties and extractability seemed unaffected. Consistently with the latter finding, *in silico* hydropathy analysis (MPEx) (Snider et al., 2009) of AZI1 and AZI1 variants carrying dephospho- or phosphomimetic exchanges at any or several of its putative phosphorylation sites predicts membrane-associated domains of identical length and position (Supplemental Figure 4).

In an attempt to assess whether lower abundance of AZI1–myc in 35::*AZI1*/*mpk3* was due to elevated degradation by the 26S proteasomal machinery, seedlings were incubated in the presence or absence of 50 μM MG132, and subsequently assessed by immunoblotting (Figure 10C). MG132 treatment (3 and 24h tested) did not induce AZI1 protein accumulation, suggesting that AZI1 protein is not a primary substrate for 26S proteasome-mediated degradation, neither in the wild-type *MPK3* ^+/+^ nor in the *mpk3* ^−/−^mutant background.

### MPK3 Controls Endogenous and Transgenic *AZI1* Transcript Levels

**Figure 11.**
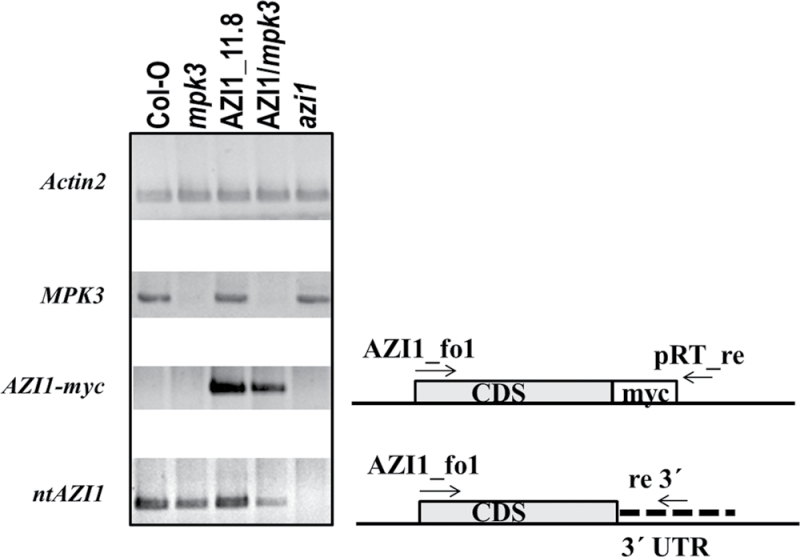
Seedlings of the Indicated Lines Were Grown on Sterile Medium. Total RNA was isolated from leaves of 11-day-old plants. Primers for semi-quantitative RT–PCR were positioned to distinguish between transgene-derived (AZI1–myc) and endogenous, non-transgenic (nt) *AZI1* expression.

It is a known phenomenon that transgene expression can be progressively lost during seed propagation. To address this aspect, 35S::*AZI1*/ and 35S::*AZI1*/*mpk3* seedlings were analyzed by RT–PCR. The reverse primer was positioned downstream of the myc-encoding region in order to selectively amplify transgene-derived *AZI1* cDNA. A PCR product was obtained from the transgenic lines, but not from the Col-O control (Figure 11). Compared to 35S::*AZI1*, transcript levels of *AZI1–myc* were slightly reduced in 35S::*AZI1/mpk3.* This may partially explain why 35S::*AZI1/mpk3* lines contained less AZI1–myc protein. Reduced transcript abundance might be attributable to (1) non-specific effect as a result of seed propagation, (2) an overall lower 35S promoter activity in *mpk3*, (3) reduced AZI1 transcript stability. Scenario (1) appears unlikely, since 35S::*AZI1/MPK3* ^*+/+*^ lines that had undergone the entire crossing and seed propagation procedure contain similar AZI1–myc protein levels as the 35S::*AZI1* parental line (Supplemental Figure 2). Also, the only moderate reduction of the transgene-derived *AZI1* mRNA in 35S::*AZI1/mpk3* compared to 35S::*AZI1/MPK3* unlikely explains the rather drastic difference of AZI1–myc protein levels. Scenario (2) is not supported by current literature (see the ‘Discussion’ section). To assess scenario (3), RT–PCR analyses were conducted using primers that selectively amplify non-transgenic AZI1 transcript (‘*ntAZI1*’). (The reverse primer is located in the 3′UTR.) Compared to Col-O, *ntAZI1* levels are reduced both in *mpk3* and in 35S::*AZI1/mpk3.* Expectedly, there is no product detectable in the *azi1* mutant line. *ntAZI1* levels in 35S::*AZI1* are similarly high as in Col-O, indicating the absence of an additional feed-forward mechanism (where ectopic AZI1–myc might induce endogenous *AZI1* expression). In summary, these data point to an additional role of MPK3 as positive regulator of *AZI1* at the transcript level.

## DISCUSSION

This study, for the first time, implies a LTP-related HyPRP as a direct component in the MAPK-mediated stress response. In vitro kinase assays had verified our *in silico* analyses-based starting hypothesis about a relation between AZI1 and MPK3. The stimulus-dependent expression and activity profiles of AZI1 and MPK3 had pointed to an involvement of these components in stress-related processes. Here, we have focused on responses of *Arabidopsis* to high-salinity conditions.

Overexpression of *AZI1* markedly improved salt-stress tolerance, both during germination and at the seedling stage (Figures 7–9). The hypersensitivity exhibited by *azi1*-null mutants furthermore suggests that AZI1 has a non-redundant function under high-salinity conditions. This adds to the previously reported role of AZI1 in cold stress protection (Zhang and Schlappi, 2007). In their salt-stress hypersensitivity, *azi1* closely resemble *earli1* mutants (Xu et al., 2011a). Apparently, neither *AZI1* nor *EARLI1* deficiency can be fully compensated for by other members of the EARLI or LTP family. Similarly to *EARLI1* overexpression (Xu et al., 2011a), elevated *AZI1* levels improve salt tolerance. Consequently, hypersensitivity in the mutants is not merely due to an imbalance in the relative amounts of EARLI-type HyPRPs. Whether ectopically expressed *AZI1* or *EARLI1* contribute interchangeably to reaching a certain tolerance-improving threshold would have to be assessed through overexpression of *AZI1* in *earli1* mutants or vice versa.

We studied the relation between AZI1 and MPK3 by overexpressing *AZI1* in the *mpk3* mutant background. 35S:*:AZI1* (line 11.8) was crossed with *mpk3* to generate lines (35S::*AZI/mpk3*) that are homozygous for the *AZI1* transgene and the *mpk3* allele. The salt-stress hypersensitivity in *mpk3* was partially overcome by overexpression of the *AZI1* transgene (Figures 8 and 9). However, functional MPK3 appears to be required for AZI1 to reach its full protecting function: 35S::AZI/*mpk3* show Col-O wild-type tolerance levels, but 35S::AZI/*MPK3* are markedly more robust. Possible explanations for this phenomenon are AZI1 needs to bind to and/or be phosphorylated by MPK3 to become fully functional. Since *AZI1* overexpression did improve tolerance in *mpk3* to some extent, an alternative MAPK, such as the closely related MPK6, may functionally replace MPK3 in this constellation. We did observe MPK6–AZI1 binding in vitro (Figure 2). Alternatively, even in the absence of phosphorylation or MAPK interaction, AZI1 may have some basal stress-protectant activity which would further increase in the presence of MPK3. It will be intriguing to see whether modification of AZI1 by MPK3 occurs in a stress-dependent manner. This aspect was not addressed here, and it clearly requires substantial additional research.

Members of the EARLI family contain a soluble 8-Cysteine-Motif and non-soluble PRD whose function might be in binding to the membrane and cell wall, respectively (Zhang and Schlappi, 2007). Consistently with this, AZI1 was found in the fraction of organic solvent extracts from *Arabidopsis* plasma membranes (Mitra et al., 2009). Immunoblot analyses of the insoluble and soluble fraction plus an additional protein extraction procedure revealed similar relative distribution of the *AZI1* transgene product in 35S::*AZI1* and 35S::*AZI*/*mpk3* lines (Figure 10B). *In silico* studies corroborate that AZI1 localization is likely independent of the protein’s phospho-status: When replacing the five putative MAPK-phosphorylation target residues (S33, 41, 59, T6, T70; see Figure 1) by alanine or aspartic acid, to generate constitutively (de)phosphomimetic AZI1 variants, similar hydropathy blots are obtained for the resultant sequences (Supplemental Figure 4).

Protein levels of ectopically expressed *AZI1* were consistently lower in 35S::*AZI1*/*mpk3* compared to 35S::*AZI1*/*MPK3* plants, irrespective of plant age. This effect is unlikely due to a general impairment of the translational machinery in *mpk3* (Supplemental Figure 3). Also, as exemplified in a recent overexpression study of *WAK2*, a receptor-like wall-associated kinase (Kohorn et al., 2012), the *CaMV35S* promoter is equally active in wild-type and *mpk3* mutant plants. RT–PCR data indicate *AZI1* transcripts to be destabilized in the *mpk3* mutant background. The altered abundance in AZI1–myc transcripts in 35S::*AZI1* versus 35S::*AZI1*/*mpk3* lines may only partially explain the differences of AZI1–myc protein in these lines. It appears likely that MPK3 controls AZI1 both at the transcript and the protein level. AZI1 protein seems to be less stable in *mpk3*—an assumption that is supported by *in silico* data: according to bioinformatic algorithms, AZI1 is a predicted unstable protein (ExPaSyProtParam tool). Upon successive replacement of the putative MAPK target sites (S33, S41, S59, T66, T70) by phosphomimetic residues (aspartate, D), the instability index of the resultant theoretic proteins gradually declines. AZI1 variants that carry three or more phosphomimetic residues are classified as stable (Supplemental Figure 5).

The mechanism regulating AZI1 protein stability is still unclear. Treatment with MG132 had no discernible effect on AZI1 protein abundance (Figure 10C). A major regulation of AZI1 by 26S-proteasome-mediated degradation therefore seems unlikely. AZI1 breakdown might follow a distinct pathway that is accelerated in *mpk3*. For instance, *bri1-5*, a defective variant of the BRI1 receptor, is effectively degraded by a proteasome-independent endoplasmic reticulum-associated degradation (Hong et al., 2008). That MAPK can control their targets at the level of protein stability has been demonstrated for ACS2/ACS6, rate-limiting enzymes in ethylene biosynthesis (Liu and Zhang, 2004). Phosphorylation by MPK6 reduced the turnover of ACS6 by the ubiquitin proteasome machinery (Joo et al., 2008).

An alternative or additional explanation for the differences in AZI1 protein levels is that *mpk3* lacks as-yet unknown other biochemical factors that stabilize the AZI1 protein. Such factors could, for example, be proteins that bind to and/or are phosphorylated by MPK3 or metabolites whose synthesis is impaired in *mpk3*. The nature of the moietie(s) accounting for the retarded migration of AZI1 in SDS–PAGE analyses is unknown. The availability and/or transfer of such moietie(s) might be a limiting step in AZI1 protein accumulation—a step at which *mpk3* is compromised. So far, the classification of AZI1 as ‘HyPRP and LTP-related protein’ is based on amino acid sequence data only. Whether MPK3 only controls AZI1 abundance or whether it modifies additional protein properties (that are not discernible by SDS–PAGE), such as enzymatic activity, remains an intriguing question for future research. There are few known (Rodriguez et al., 2010) and certainly numerous elusive factors contributing to MPK3-mediated stress tolerance. The finding of 35S::AZI/*mpk3* being less tolerant than 35S::AZI/*MPK3* plants most likely also derives from impaired posttranslational modification of further MPK3 target proteins, such as MYB44 (Persak and Pitzschke, 2013), in the former line. The salt-hypersensitive phenotype of *mpk3* might, at least partially, be attributable to a limited availability of AZI1. This assumption is supported by the findings that *AZI1* transcript levels are reduced in *mpk3* and that the salt tolerance of *mpk3* can be improved by ectopic expression of AZI1.

Immunoblot analyses of 35S::*AZI1* and 35S::*AZI1/mpk3* plants revealed quantitative, but no qualitative, differences (Figure 10A). In both cases, AZI1–myc protein migrated as a 36/38-kDa double band, which is significantly higher (>6kDa) than expected. In contrast, gel migration of recombinant AZI1 peptides isolated from *E. coli* was normal. These observations suggest AZI1 to undergo some posttranslational, eukaryotic-specific modification, which is seemingly MPK3-independent. A likely modification that could account for this comparatively large size difference is glycosylation. Glycoproteins are synthesized on the rough ER, followed by the addition of sugar units in the cisternae of the ER. Glycosylated proteins are then carried to the plasma membrane, where they are incorporated or secreted. Several AZI1 properties support this ‘glycosylation hypothesis’:

(1) eukaryotic-specific retarded gel migration (this study);
(2) presence of secretion signal;
(3) reported AZI1 localization (ER; Yu et al., 2013) and plasma membrane (Zhang and Schlappi, 2007);
(4) sequence similarity with known glycoproteins (see below).

Of the two known types of glycosylation (O- or N-), only O-glycosylation is principally possible. The AZI1 protein sequence is devoid of any Asn-X-Ser/Thr motif, the target site for N-glycosylation (Wilson, 2002). Bioinformatic algorithms (NetOGlyc) predict nine possible O-glycosylation sites, positioned at serine residue 33, 41, 56, 59, 66, 73, 74, 77, and threonine residue 66, 70 (Supplemental Figure 6). In addition, based on sequence homology and the composition of the proline-rich-domain in AZI1, also O-Hyp glycosylation (addition of sugar units at hydroxy-proline residues) appears plausible. Clustered, non-contiguous Hyp residues, matching the motif X-Pro-X-Pro-X-Pro have been shown to direct arabinogalactan heteropolysaccharide addition in *Arabidopsis* and tobacco (Xu et al., 2008). Size fractionation of base hydrolysates of plant arabinogalactan proteins (AGPs) and a synthetic gene encoding Ser-Pro-Ser-Pro-Ser-Pro repeats showed two distinct size populations (Xu et al., 2008), reminiscent of the double band that we observed for AZI1–myc. The contiguous span of eight X-Pro repeats contained in the AZI1 protein sequence (residues 31–46) further qualifies AZI1 as candidate O-glycoprotein. In its gel migration properties and composition of the PRD, AZI1 resembles AGP31 (Arabionogalactan protein 31) from *Arabidopsis* (Hijazi et al., 2012). MALDI–TOF analyses had disclosed an extensive and heterogenous glycosylation pattern for AGP31. Three fragments within the PRD of AGP31 are verified glycopeptides (Hijazi et al., 2012). As displayed in the alignment (Supplemental Figure 7), substantial homology exists between AZI1 and AGP31 tryptic peptide P4, the most heavily glycosylated peptide reported by Hijazi et al. (2012). A characteristic feature of glycoproteins is their ability to form insoluble complexes with Yariv reagents (Smallwood et al., 1996). However, using Yariv for confident analysis is somewhat problematic: Yariv-reactivity, for unknown reasons, does not always correlate with positive glycosylation, nor is it proportional to the glycan length (Xu et al., 2008). Furthermore, some proteins are recognized by the Yariv reagent, yet they show no lectin binding—another characteristic of glycoproteins (Hijazi et al., 2012). Given these complications, we have limited research on a possible AZI1 glycosylation to *in silico* analysis only. In conclusion, several properties of AZI1 support the assumption of O-glycosylation. Future research shall test this hypothesis and elucidate the type, position, and length of (the hypothesized) glycan moieties in the AZI1 protein.

Co-immunoprecipitation and BiFC experiments in transgenic plant tissue (Figures 5 and 6) are supportive of an AZI1/MPK3 in vivo interaction. Future experiments with plant material collected at several developmental stages, after stress exposure of various intensity and duration, shall elucidate the dynamics and environmental dependency of complex formation *in planta.* Interestingly, BiFC studies in *Arabidopsis* mesophyll protoplasts transiently expressing AZI1–cYFP/MPK3–nYFP yielded no positive signal. These experiments were repeated several times and technical functionality of the assay was verified by including a known pair of interactors, MKK4–cYFP/MPK3–nYFP (Pitzschke and Persak, 2012), as positive control. A possible explanation is that a cell–cell contact is required for the physical association of AZI1 and MPK3. In this respect, it is interesting to note that the BiFC signals seen in leaf tissue (Figure 5) appear to derive predominantly from the contact sites of adjacent cells. This property would be in favor of a plasmodesmal localization and thus be in good agreement with Yu et al. (2013), who also detected AZI1 in plasmodesmata. At this stage, we cannot confidently assign the protein complex to a certain cellular compartment. An exclusive plasmodesmal location seems unlikely because in that case a more punctuate pattern (see, e.g. Yu et al., 2013) would be expected.

The distribution of AZI1/MPK3 association is distinct from that of MKK4/MPK3 complexes (cytoplasm and nucleus) (Persak and Pitzschke, 2013; Pitzschke, 2013). By comparing Figures 4 and 5, it can furthermore be concluded that only a certain fraction of MPK3 is recruited for the interaction with AZI1. More detailed studies, including a series of marker proteins, will be required to confidently identify the subcellular compartment of AZI1/MPK3 complexes.

Apart from the involvement of MPK3 and AZI1 in the salt-stress response demonstrated here, the two proteins have been—independently—correlated with biotic stress (Beckers et al., 2009; Jung et al., 2009). Both mutants, *azi1* and *mpk3*, are compromised specifically in systemic acquired resistance (SAR). One possible reason for the SAR-deficient phenotype in *mpk3* could be the incapability of generating/maintaining sufficient amounts of functional AZI1 protein. Unlike this hypothetical direct control of AZI1, MPK3 may not or only indirectly regulate the LTP DIR1. Similarly to *azi1* and *mpk3*, *dir1* mutants are compromised in SAR (Maldonado et al., 2002). However, the DIR1 protein sequence is devoid of a kinase interaction domain (Figure 1). Only recently, a pioneering study had established a direct link between AZI1 and DIR1 as cooperate mediators of SAR (yu). The authors disclosed a sophisticated feedback regulatory loop between these two LTPs and glycerol-3-phosphate (G3P), a mobile sugar derivative. While exogenously applied G3P induced AZI1 and DIR1 accumulation, overexpression of AZI1 or DIR1 led to an increase in G3P levels. AZI1 and DIR1 were found to form homo- and heterodimers, and their overlapping localization in ER and plasmodesmata further supports the idea that one of these proteins may function as a unit. In light of the known implication of MPK3 in SAR (Beckers et al., 2009) and the lowered abundance of AZI1 in *mpk3* mutants (this study), some intriguing questions arise:

(1) Does G3P trigger a raise in MPK3 protein and/or kinase activity, which would in turn support AZI1 synthesis and stability?
(2) Are *mpk3* mutants SAR-deficient because they fail to accumulate AZI1, to produce, sense, or to transport G3P?
(3) Given the fact that G3P induces AZI1 accumulation, and that AZI1 acts as positive regulator under salt stress, is G3P sufficient and active to improve salt-stress tolerance in plants? Would such effect be limited to *Arabidopsis*?
(4) Given the systemic effect of G3P (in the pathogen response), is pre-treatment of young plants sufficient to fortify them against environmental stresses encountered at a later stage?

In a global homology study of cysteine-rich proteins (CRPs) in *Arabidopsis* and rice, Silverstein et al. (2007) observed that highly similar CRPs have expanded in tandem arrays within each genome. However, there is one subgroup, CRP480, named ‘Proline-rich protein-LTPs; PRP-LTPs’ that is highly conserved between the two species (Silverstein et al., 2007). This very subgroup is identical to the HyPRP family. The *EARLI* gene cluster as well as five of the 12 multiple-stress-responsive *LTP* genes (Supplemental Table 1) are contained in this particular subgroup. In light of the strong conservation of MAPK signaling mechanisms, it is tempting to speculate that HyPRP–MAPK associations are a universal scheme employed in plant stress adaptation. So far, LTPs had not been directly connected with MAPKs. How the AZI1/MPK3 association relates to known osmotic stress response pathways remains obscure at this stage. Other MAPK signaling modules, particularly the MKK1/MKK2–MPK4 relay that mediates salt and cold stress signaling in *Arabidopsis* (Teige et al., 2004), are at least indirectly linked with LTPs: transcriptome profiling had revealed a number of LTP-encoding genes (other than *EARLI*s) to be differentially regulated in *mkk1/mkk2* and/or *mpk4* mutants (Qiu et al., 2008; Pitzschke et al., 2009a).

Interestingly, overexpression of *EARLI* genes confers resilience to freezing and osmotic stress in yeast, an organism that naturally lacks LTPs (Zhang and Schlappi, 2007). Similarly to the situation in plants (Andreasson and Ellis, 2010; Sinha et al., 2011), these abiotic stresses are associated with MAPK activation also in yeast (Panadero et al., 2006; Zi et al., 2010). Future studies shall help to clarify whether *Arabidopsis* EARLI proteins are recognized and modified by stress-induced yeast MAPKs to reach their full protecting activity. In engineered plants, the targeted exchange of critical residues (phosphorylation sites; MAPK binding sites) may be a means to manipulate reported HyPRP-controlled processes such as fruit ripening (Blanco-Portales et al., 2004), fungal growth inhibition (Li et al., 2012), or freezing tolerance (Zhang and Schlappi, 2007; Xu et al., 2011b). Based on its gene expression profile (induced by numerous stressors), functions of AZI1 as a protectant towards other types of stress seem likely. This could also be of agricultural interest, particularly since—in contrast to the retarded growth frequently observed in stress-resistant mutant or transgenic plants (Ezhova et al., 2001; Magome et al., 2004; Suarez-Rodriguez et al., 2007; Qiu et al., 2008; Krishnaswamy et al., 2011; Tsugama et al., 2012)—transgenic AZI1 plants develop normally.

## METHODS

Accession numbers: AZI1 At4g12470; MPK3 At3g45640; MPK6 At2g43790.

### In Vitro Pull-Down Assay

AZI1 recombinant proteins were produced using the intein-splicing system (IMPACT™, Biolabs). *E. coli* strain BL-21 DE3 was transformed with vector pTwin, containing AZI1 coding sequences (PRD: residues 25–72; PRD-8CM: residues 25–end). Proteins of IPTG-induced cultures were extracted and immobilized on a chitin matrix according to the manufacturer’s instructions. GST–MPK3 or GST–MPK6 fusion proteins were expressed in BL-21 DE3 as described previously (Djamei et al., 2007). Immobilized AZI1 peptides were incubated with protein extracts from GST–MPK-expressing *E. coli*. Captured proteins were separated via SDS–PAGE and visualized by Coomassie Blue staining or immunoblotting using mouse anti-GST and horse-radish-peroxidase-conjugates rabbit-anti-mouse antibodies.

### In Vitro Kinase Assay

Tag-free AZI1 peptides were purified from *E. coli* cell cultures (see above) according to the manufacturer’s instructions. GST–MPK3 fusion protein was expressed and purified as described previously (Djamei et al., 2007). In vitro kinase assays with gamma-^32^P-labelled ATP, purified GST–MPK3 and AZI1 proteins were performed in kinase reaction buffer 20mM HEPES pH 7.4; 15mM MgCl_2_; 5mM EGTA; 1mM DTT) at room temperature for 1h. After addition of protein loading, buffer and heat-denaturation samples were separated by 15% SDS–PAGE. Vacuum-dried gels were exposed to X-ray film (Amersham).

### Plasmids for Plant Transformation

35S::AZI1–myc and 35S::MPK3–YFP pGreen constructs for overexpression in plants were generated by replacing the MYB44 coding sequence in the previously described plasmids 35S::MYB44–myc or 35S::MYB44-YFP (Persak and Pitzschke, 2013), respectively. A construct for the CaMV35S-driven expression of AZI1 fusions to the C-terminal part of YFP (AZI1–cYFP) was generated by replacing the MKK4 coding sequence in 35S::MKK4–cYFP (Persak and Pitzschke, 2013) with that of AZI1. Plasmid integrity was verified by restriction digest and sequencing. Verified plasmids were subsequently transformed into *Agrobacterium tumefaciens* GV3101 carrying the helper plasmid pSOUP via electroporation. Kanamycin-resistant positive transformants were further verified by colony–PCR.

### Plant Material, Crossing, and Stress-Tolerance Tests

All *Arabidopsis thaliana* lines used are in the Columbia-0 background. These include Col-O wild-type, *mpk3* (SALK_151594), and *azi1* (SALK_017709; accession number N517709). Plants were routinely grown at 20°C, 60% humidity under long-day conditions (16/8-h light/dark). Col-O plants were transformed with a CaMV35S::AZI1–myc construct via the floral dip method (Clough and Bent, 1998). Transgenic seeds were selected and propagated to homozygosity on half-strength MS medium supplemented with 1% agar, 0.25% sucrose, and 50 μg ml^–1^ kanamycin. Transgene expression was verified by immunoblot analysis.

A homozygous 35S::AZI1–myc line (‘#11.8’) (pollen) showing strong transgene expression was crossed into the *mpk3* mutant background (pistil). T3 progeny homozygous for both the *mpk3* allele and the transgene was identified by kanamycin resistance and genotyping. Homozygous mutant and T3 transgenic lines were used for the stress assays. In an initial screen, enhanced salt-stress tolerance was observed for all independent 35S::*AZI1*–myc lines tested. To allow direct comparison between 35S*::AZI1* and 35S:*:AZI1/mpk3*, only the corresponding parental line (*35S::AZI1_11.8*, pollen donor) was used. For 35S:*:AZI1/mpk3*, at least three sublines were included in each analysis. The seeds were of similar age (11–15 months old), had been harvested from different batches of plants (grown under identical conditions), and had been stored in the same place. All stress-tolerance tests were independently repeated at least three times, using 40–50 seeds per experiment per line. Seeds were surface-sterilized with NaOCl/96% ethanol for 5min and washed three times with 96% ethanol. Surface-sterilized seeds were incubated on standard medium (half-strength MS medium/0.25% sucrose/1% plant agar) with or without 150mM NaCl. After stratification for 2 d at 4°C, seeds were incubated at 25°C under a 8/16-h dark/night regime. Germination percentage was monitored over a 5-day period. Seeds were considered ‘germinated’ after radicles had emerged. To assess post-germination stress tolerance, seeds were plated on standard medium (see above). After 3 d, seedlings were transferred to plates standard medium supplemented with 250mM NaCl. Survival was monitored over a 3-day period.

### Protein Extraction and Immunoblot Analysis

*Arabidopsis* seedlings were shock-frozen and stored at –80°C or directly pulverized under liquid nitrogen. An equal volume of buffer (50mM Tris/HCl, pH 7.5, 5mM EDTA pH 8, 5mM EGTA pH 8, 2mM DTT, 100mM β-glycerophosphate, 10mM Na-Vanadat, 10mM Na-Fluorid, 10mM PMSF, 10 μg ml^–1^ aprotinin, 10 μg ml^–1^ leupeptin) was added to the powder, and samples were incubated for 10min on ice. The non-soluble fraction was removed by centrifugation of samples at 4°C, 14000 *g*, 15min. Protein concentrations of supernatant fluid was determined by the Bradford assay (BioRad), using BSA as standard. Samples were denatured in 1 SDS-loading dye for 5min at 95°C. 15 μg protein were separated by SDS–PAGE and blotted on a PVDF membrane (Porablot, Roth). The non-soluble fraction was denatured directly. The chemical lysis procedure (Tsugama et al., 2011) was applied to an independent set of seedlings (five or six seedlings per sample, equal weight) and processed for immunoblot analysis. For immunodetection of AZI1–myc fusion proteins, rabbit anti–myc and IRDye800CW-coupled donkey-anti-rabbit (NEB Biolabs) were used as primary and secondary antibodies, respectively. Membranes were scanned with a LiCOR Odyssee machine, at 800nm for the detection of bound antibodies.

### Co-Immunoprecipitation

Proteins were extracted *N. benthamiana* agro-infiltrated leaves (5 dpi) in buffer N (100mM Tris pH 7.5, 75mM NaCl, 1mM EDTA, 1mM EGTA, 0.1% triton, 0.05% SDS, 10% glycerol, 2.5mM DTT, 0.5mM PMSF, 5 μg ml^–1^ leupeptin, 5 μg ml^–1^ aprotinin, 5mM Na-Fluorid, 5mM Na-vanadate. Protein concentrations in the supernatant liquids obtained after 15-min centrifugation (4°C, 14000 *g*) were adjusted to 2 μg μl^–1^ (Bradford assay) by adding the respective volume of bufferN. A 200-μl sample was incubated with 1 μl anti-MPK3 antibody at 4°C overnight on a rotating wheel. MPK3 protein complexes were captured by adding 5 μl Dynabeads ProteinA (Invitrogen) (equilibrated in TBS) and incubation of the samples for another 3h at 4°C. Beads were collected using a magnetic rack and washed three times in 200 μl buffer N. After the final wash, samples were transferred into a fresh 1.5-ml tube and finally re-suspended in 30 μl 1 protein loading dye. Following heat-denaturation, 7-μl sample volume was separated on a 12% SDS–PAGE. Immunoblot analysis was performed as described above.

### MPK3 Localization and BiFC Studies in *Tropaeolum majus* and *Nicotiana benthamiana*

*Agrobacterium tumefaciens* GV3101 carrying the helper plasmid pSOUP and pGreen derivatives (CaMV35S::YFP; CaMV35S::MPK3–YFP; CaMV35S::AZI1–cYFP; CaMV35S::MPK3– nYFP) were grown at 28°C on LB agar supplemented with rifampicin, tetracyclin, and kanamycin. *Agrobacterium* suspensions were adjusted to OD 0.2 in 10mM MES (pH 5.7), 10mM MgCl_2_, and 100 μM acetosyringone and incubated for 3h prior to leaf infiltration. *Agrobacterium*-mediated transformation of *T. majus* and *N. benthamiana* has been described recently (Persak and Pitzschke, 2013; Pitzschke, 2013). For BiFC experiments, leaves were co-infiltrated with agrobacterial suspensions of AZI1–cYFP and MPK3–nYFP. Co-infiltrations of [AZI1–cYFP/nYFP empty vector] or [MPK3–nYFP/cYFP empty vector] served as negative controls. For MPK3–YFP localization studies in *N. benthamiana*, pieces of agro-infiltrated (4 dpi) leaves were either used directly or after floating on 2 M NaCl for 2–8min. Subcellular localization studies were conducted 3–5 d post infiltration using a UV microscope (Leica DM5500B).

### Semi-Quantitative RT–PCR

Leaves from 14-day-old seedlings (grown on the same plate to minimize position effects; three replicates) were directly collected in liquid nitrogen. The tissue was pulverized and total RNA was extracted (Trizol reagent). Samples were DNase I-digested and reverse-transcribed (Fermentas) as described previously (Pitzschke et al., 2009b). The following primer combinations were used for PCR amplification: Actin2_fo1/re1 (At2g37620 CDS; atggttaaggctggttttgc/agcacaataccggtagtacg), AZI1_fo1 (CDS start; acccatggcttcaaagaactcagc/pRT_re (plasmid-derived sequence; gggaactactcacacattat), AZI1_fo1 (CDS start; acccatggcttcaaagaactcagc)/AZI1_re7 (3′UTR; ggggacaacgtttacaaacaa), and MPK3_fo/re (At3g45640 CDS; ggtctgttggttgtatcttt/agatactaagtagccattcg). Amplification products were separated on 1.2% TAE agarose gels and visualized by EtBr staining.

## SUPPLEMENTARY DATA

Supplementary Data are available at *Molecular Plant Online.*

## FUNDING

Research was funded by grants of the Austrian Science Foundation (FWF), V167-B09 (Elise-Richter-Project to A.P.), and P21951-B09.

## Supplementary Material

Supplementary Data
