# Supplementary material for: Salt Stress in Arabidopsis: Lipid Transfer Protein AZI1 and Its Control by Mitogen-Activated Protein Kinase MPK3
Source: Mol Plant. 2013 Nov 8;7(4):722–38. doi: 10.1093/mp/sst157 (PMC3973493; doi:10.1093/mp/sst157)
Supplement: Supplementary Data [file supp_7_4_722__index.html]

Salt stress in Arabidopsis: Lipid transfer protein AZI1 and its control by Mitogen-activated protein kinase MPK3 — Salt Stress in Arabidopsis: Lipid Transfer Protein AZI1 and Its Control by Mitogen-Activated Protein Kinase MPK3 — Salt Stress in Arabidopsis: Lipid Transfer Protein AZI1 and Its Control by Mitogen-Activated Protein Kinase MPK3 — Supplementary Data 

# Salt Stress in *Arabidopsis*: Lipid Transfer Protein AZI1 and Its Control by Mitogen-Activated Protein Kinase MPK3

## Supplementary Data

Data files

**Files in this Data Supplement:**

- Supplementary Data - Supplementary Data
