# Supplementary material for: Salt Stress in Arabidopsis: Lipid Transfer Protein AZI1 and Its Control by Mitogen-Activated Protein Kinase MPK3
Source: Mol Plant. 2013 Nov 8;7(4):722–38. doi: 10.1093/mp/sst157 (PMC3973493; doi:10.1093/mp/sst157)
Supplement: Supplementary Data [file supp_sst157_MPLANT20130022046_suppl_data.pdf]

Supplementary data

Suppl. figure S1

Confirmation of knock-out *azi1* mutant by RT-PCR.

Total RNA was isolated from 14-day-old seedlings. cDNA samples were amplified with primers for *Actin2* (control) or for *AZI1*.

Suppl. figure S2

Lower AZI1 levels in *35S::AZI1/mpk3* lines are related to the *mpk3* mutant background.

Proteins were extracted from 14-day-old seedlings originating from crossings of the *AZI1* overexpressing line 11.8 with *mpk3*. AZI1-myc protein was detected by immunoblot analysis after SDS-PAGE. Protein loading was visualised by Coomassie-blue staining. AZI1 X1 and X2 are wildtype for *MPK3*, while AZI1/*mpk3* (two independent lines shown) lack a functional *MPK3* allele. X1 and X2 have undergone the same crossing and seed propagation procedure as AZI1/*mpk3*.

Suppl. figure S3

The overall protein profiles in Arabidopsis wild-type, *azi1*, *mpk3*, 35S::*AZI1* and 35S::*AZI1*/*mpk3* plants look similar.

Proteins from 14-day-old seedlings of the indicated lines (independent duplicate samples) were extracted in standard extraction buffer B1. 15 µg of the soluble protein fraction was separated by SDS-PAGE and visualised by Coomassie Blue staining.

Suppl. figure S4

Hydrophathy profile of AZI1 and variants carrying dephosphomimetic (alanine, A) or phosphomimetic (aspartate, D) residues at putative MAPK phosphorylation sites (Ser33, Ser41, Ser59, Thr66, Thr70).

Data were computed for the full-length AZI1 protein sequence with or without one of the following replacements:

„AAA“: S33A,S41A,S59A

„DDD“: S33D,S41D,S59D

„AAAAA“:S33A,S41A,S59A,T66A,T70A

„DDDDD“: D,S41D,S59D,T66D,T70D

The **black curve** is the actual profile; the superimposed green curve is a smoothed version of the profile.

Regions that have a favorable free energy of transfer (predicted membrane-located regions) are indicated with horizontal red bars.

Suppl. figure S5

Predicted stability of AZI1 and phosphovariant proteins.

Sequences were submitted to the ExpasyProtParam tool. The computed instability index (II) inversely correlates with protein stability. Proteins with II<40 are predicted as stable. Note the decline in II upon successive exchange of putative phosphorylation sites by phosphomimetic aspartate (D) but not by dephosphomimetic alanine (A) residues.

Suppl. figure S6.

Predicted O-glycosylation sites in AZI1.

The full-length AZI1 protein sequence was analysed using the bioinformatic online tool NetOGlyc, according to which "scores higher than 0.5 are predicted as glycosylated and marked with the string "#POSITIVE" in the comment field". The relevant region comprising all predicted sites is displayed at the bottom. Nine candidate target sites for O-glycosylation (blue) are contained in the proline-rich domain of AZI1. Five of these (Ser33, Ser41, Ser59, Thr66, Thr70) overlap with putative MAPK target sites (red) (see also figure 1).

Suppl. figure S7

Alignment of *Arabidopsis* AGP31 sequence of residues 98-129, a reportedly strongly glycosylated peptide (Hijazi et al., 2012), with the proline-rich domain of AZI1. Data were generated using the PRALINE software http://www.ibi.vu.nl/programs/pralinewww/.

Table S1. Multiple stress-responsive LTPs

Arabidopsis *LTP* genes responding to 6 or more types of abiotic stress, as identified in a functional genomics-based screen (Kant et al. 2008).*Corresponding subfamily of cysteine-rich proteins (CRPs). Members of CRP4820, the only subfamily with strong conservation between monocots and dicots (Silverstein et al. 2007) are highlighted.

Table S2 Statistic evaluation of data from germination and seedling survival assays

Table S1. Multiple stress-responsive LTPs

Arabidopsis *LTP* genes responding to 6 or more types of abiotic stress, as identified in a functional genomics-based screen (Kant et al. 2008).

*Corresponding subfamily of cysteine-rich proteins (CRPs). Members of CRP4820, the only subfamily with strong conservation between monocots and dicots (Silverstein et al. 2007) are highlighted.

LTP (PRD-8CM)	Subfamily*
AT5G59320	CRP3860
AT5G59310	CRP3860
AT3G51600	CRP3860
AT3G43720	CRP4540
AT3G22600	CRP4630
AT1G27950	CRP4640
AT2G37870	CRP4750
AT3G18280	CRP4920
AT1G62510	CRP4820
AT2G10940	CRP4820
AT2G45180	CRP4820
AT4G12470	CRP4820
AT4G22470	CRP4820

Suppl. Table S2.1: Percentage germination of seeds of indicated plant lines on ½ MS+ 0.25% at various time points

	Day 1			Day 2			Day 3			Day 4			Day 5		
Plant lines	Mean ± SD	p value*	p value #	Mean ± SD	p value*	p value #	Mean ± SD	p value *	p value #	Mean ± SD	p value *	p value #	Mean ± SD	p value *	p value #
ColO	95.60 ± 5.75	X	NS	98.18 ± 3.14	X	NS	98.78 ± 2.09	X	NS	98.78 ± 2.09	X	NS	98.78 ± 2.09	X	X
AZI1	96.26 ± 3.06	NS	X	98.44 ± 1.99	NS	X	99.48 ± 1.02	NS	X	100.00 ± 0.00	NS	X	100.00 ± 0.00	NS	X
azi1	30.91 ± 9.11	< 0.001	< 0.001	67.01 ± 8.10	0.002	<0.001	78.81 ± 8.98	0.014	0.004	83.55 ± 5.53	0.007	0.001	88.29 ± 3.59	0.007	0.001
mpk3	90.24 ± 9.39	NS	NS	96.32 ± 3.97	NS	NS	99.12 ± 1.51	NS	NS	99.12 ± 1.51	NS	NS	99.12 ± 1.51	NS	NS
AZI1 /mpk3	92.61 ± 7.23	NS	NS	100.00 ± 0.00	NS	NS	100.00 ± 0.00	NS	NS	100.00 ± 0.00	NS	.	100.00 ± 0.00	NS	-

Suppl. Table S2.2: Percentage germination of seeds of indicated plant lines on ½ MS+ 0.25% sucrose + 150 mM NaCl at various time points

	Day 1			Day 2			Day 3			Day 4			Day 5		
Plant lines	Mean ± SD	p value *	p value #	Mean ± SD	p value *	p value #	Mean ± SD	p value *	p value #	Mean ± SD	p value *	p value #	Mean ± SD	p value *	p value #
ColO	0.79 ± 1.37	X	NS	13.28 ± 5.00	X	0.037	37.70 ± 2.67	X	0.011	57.01 ± 7.14	X	0.008	72.26 ± 2.00	X	0.003
AZI1	3.24 ± 2.29	NS	X	26.03±6.42	0.037	X	64.34 ± 11.16	0.011	X	84.58 ± 9.18	0.008	X	92.18 ± 6.05	0.003	X
azi1	0.00 ± 0.00	NS	0.030	0.00 ± 0.00	0.003	< 0.001	0.71 ± 1.42	< 0.001	< 0.001	1.35 ± 1.58	< 0.001	< 0.001	6.53 ± 6.74	< 0.001	< 0.001
mpk3	0.00 ± 0.00	NS	0.063	0.00 ± 0.00	0.010	0.001	1.04 ± 1.80	< 0.001	< 0.001	3.19 ± 3.12	< 0.001	< 0.001	38.14 ± 23.96	NS	0.007
AZI1/mpk3	0.00 ± 0.00	NS	NS	0.00 ± 0.00	0.038	0.006	4.72 ± 0.39	< 0.001	0.002	20.41 ± 10.01	0.016	0.001	53.19 ± 6.08	0.013	0.002

p value * indicates the difference between means (by t-test) between *ColO* and individual lines. p value # value indicates the difference between means (by t-test) between *AZI1* and individual line. ' – ' indicates that t test could not be computed as SD of both groups is 0.00, NS*:* Not significant, SD: Standard deviation

Suppl. Table S2.3: p values indicating differences between means for mpk3 and AZI1 x mpk3 by t-test for percentage germination on ½ MS+ 0.25% sucrose + 150 mM NaCl at various time points.

	Day 1	Day 2	Day 3	Day 4	Day 5
**½ MS+ 0.25% sucrose**	NS	NS	NS	NS	NS
**½ MS+ 0.25% sucrose + 150 mM NaCl**	-	-	NS (0.073)	Borderline (0.058)	NS

‘ – ’ indicates that t test could not be computed as SD of both groups is 0.00 , NS***:*** Not significant, SD: Standard deviation

Suppl. Table S2.4 : Percentage survival of 3 day old seedlings of indicated plant lines after transfer to ½ MS+ 0.25% sucrose + 250 mM NaCl at various time points.

	12 hours			24 hours			36 hours			48 hours			60 hours			72 hours		
Plant lines	Mean ± SD	p value *	p value #	Mean ± SD	p value *	p value #	Mean ± SD	p value *	p value #	Mean ± SD	p value *	p value #	Mean ± SD	p value *	p value #	Mean ± SD	p value *	p value #
ColO	100.00±0.00	X	X	90.00±11.5	X	X	26.25 ± 4.78	X	X	13.75 ± 4.78	X	X	6.25 ± 4.78	X	X	3.75 ± 4.78	X	X
AZI1	100.00±0.00	-	X	100.00±0.00	NS	X	61.25 ± 6.29	<0.001	X	35.00 ± 12.24	0.018	X	15.00 ± 11.54	NS	X	8.75 ± 8.53		X
azi1	100.00±0.00	-	-	62.50±5.00	0.005	< 0.001	10.00 ± 14.14	NS	0.001	10.00 ± 14.14	NS	0.037	2.5 ± 5.00	NS	NS	2.5 ± 5.00	NS	NS
mpk3	100.00±0.00	-	-	76.67±15.27	NS	0.025	15.00 ± 5.00	0.029	< 0.001	10.00 ± 10.00	NS	0.035	3.33 ± 5.7	NS	NS	0.00 ± 0.00	NS	NS
AZI1/mpk3	100.00±0.00	-	-	93.33±5.77	NS	NS	26.67 ± 5.77	0.001	0.001	13.33 ± 5.77	0.038	0.038	5.00 ± 8.666	NS	NS	1.67 ± 2.88	NS	NS

p value * indicates the difference between means (by t-test) between *ColO* and individual lines. p value # indicates the difference between means (by t-test) between *AZI1* and individual line. ‘ – ’ indicates that t test could not be computed as SD of both groups is 0.00 , NS***:*** Not significant, SD: Standard deviation

Suppl. Table S2.5: p values indicating differences between means for mpk3 and AZI1 x mpk3 by t-test for percentage survival of 3 day old seedlings after transfer to on ½ MS+ 0.25% sucrose + 250 mM NaCl at various time points.

	12 hours	24 hours	36 hours	48 hours	60 hours	72 hours
**½ MS+ 0.25% sucrose + 250 mM NaCl**	NS	NS	NS	NS	NS	NS

‘ – ’ indicates that t test could not be computed as SD of both groups is 0.00 , NS***:*** Not significant, SD: Standard deviation

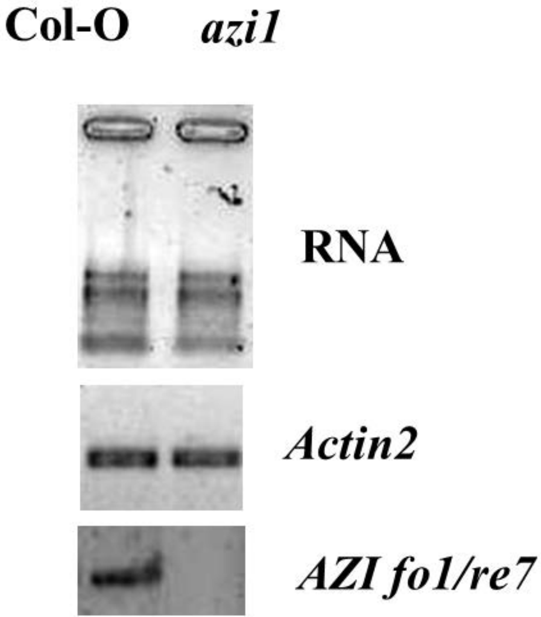

Suppl. figure S1
Confirmation of knock-out *azi1* mutant by RT-PCR.
Total RNA was isolated from 14-day-old seedlings. cDNA samples were amplified with primers for *Actin2* (control) or for *AZI1*.

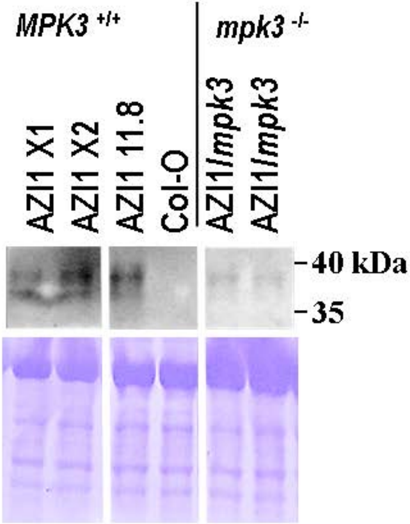

Suppl. figure S2

Lower AZI1 levels in *35S::AZI1/mpk3* lines are related to the *mpk3* mutant background.

Proteins were extracted from 14-day-old seedlings originating from crossings of the *AZI1* overexpressing line 11.8 with *mpk3*. AZI1-myc protein was detected by immunoblot analysis after SDS-PAGE. Protein loading was visualised by Coomassie-blue staining. AZI1 X1 and X2 are wildtype for *MPK3*, while AZI1/*mpk3* (two independent lines shown) lack a functional *MPK3* allele. X1 and X2 have undergone the same crossing and seed propagation procedure as AZI1/*mpk3*

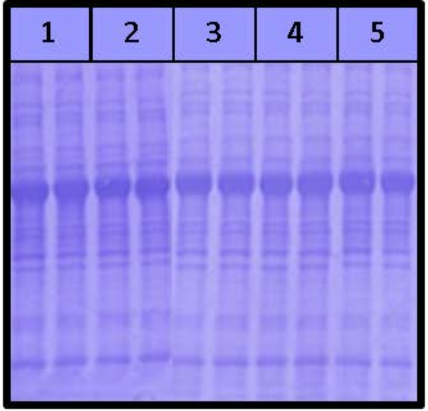

1 Col-O wild type
2 *azi1*
3 35S::*AZI1*
4 35S::*AZI1/mpk3*
5 *mpk3*

Figure S3

The overall protein profiles in Arabidopsis wild-type, *azi1*, *mpk3*, 35S-AZI1 and 35S-AZI1/*mpk3* plants look similar.

Proteins from 14-day-old seedlings of the indicated lines (independent duplicate samples) were extracted in standard extraction bufferA. 15 µg of the soluble protein fraction was separated by SDS-PAGE and visualised by Coomassie Blue staining.

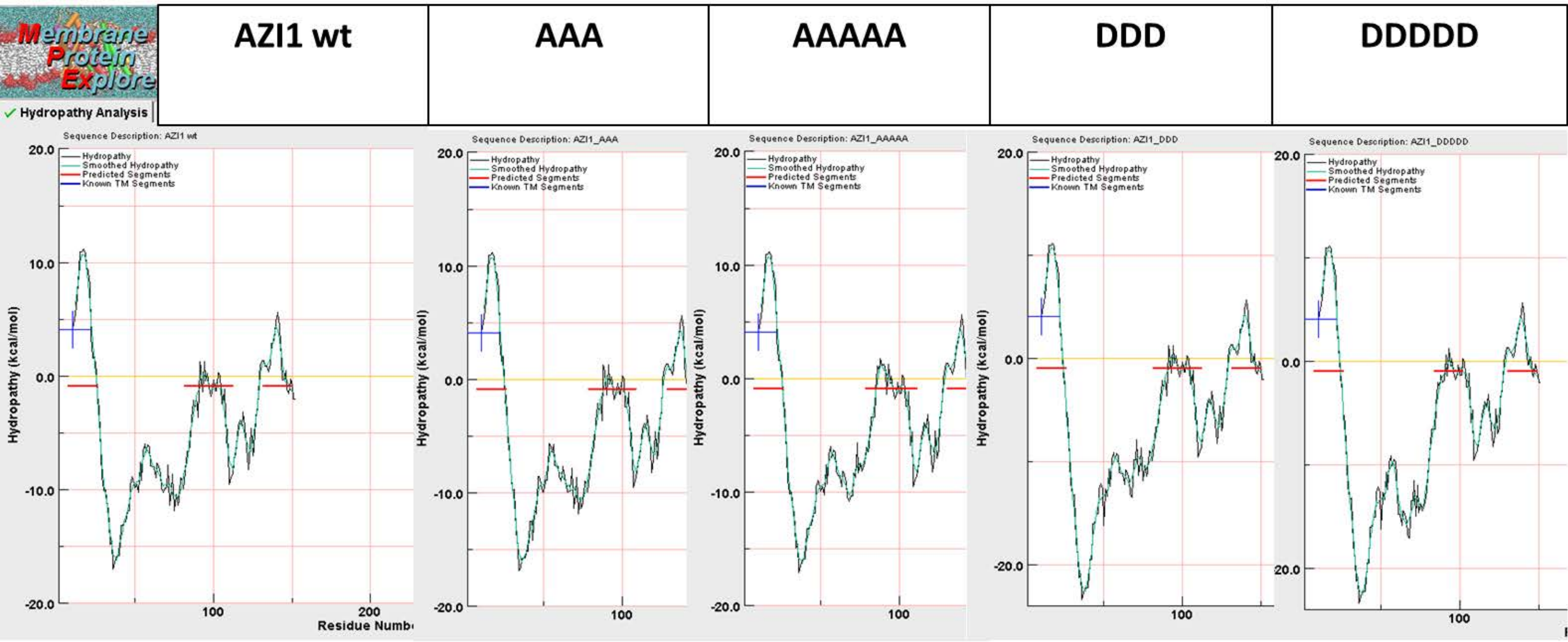

Figure S4

Hydrophathy profile of AZI1 and variants carrying dephosphomimetic (alanine, A) or phosphomimetic (aspartate, D) residues at putative MAPK phosphorylation sites (Ser33, Ser41, Ser59, Thr66, Thr70).

Data were computed for the full-length AZI1 protein sequence with or without one of the following replacements:

„AAA“: S33A,S41A,S59A

„DDD“: S33D,S41D,S59D

„AAAAA“:S33A,S41A,S59A,T66A,T70A

„DDDDD“: D,S41D,S59D,T66D,T70D

The **black curve** is the actual profile; the superimposed green curve is a smoothed version of the profile.

Regions that have a favorable free energy of transfer (predicted membrane-located regions) are indicated with horizontal red bars.

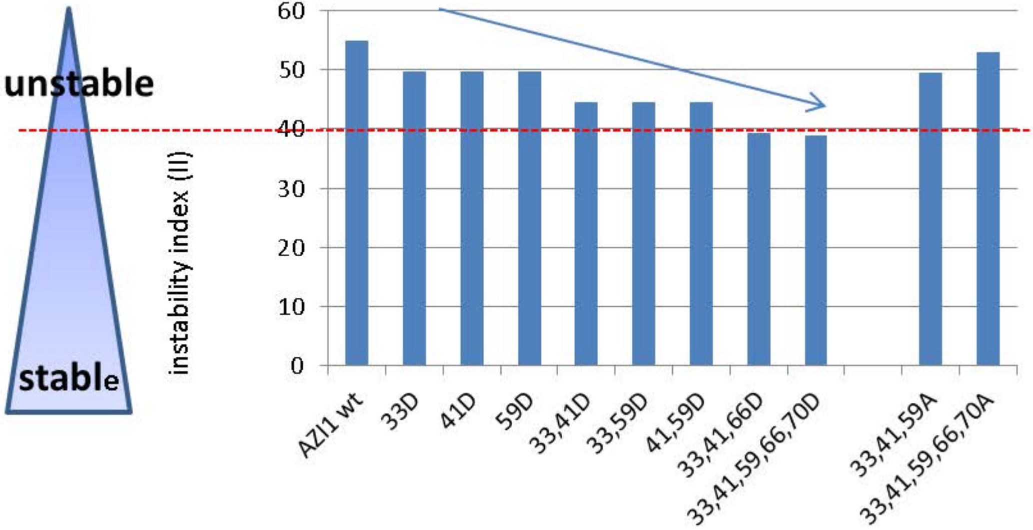

Figure S5

Predicted stability of AZI1 and phosphovariant proteins.

Sequences were submitted to the ExpasyProtParam tool. The computed instability index (II) inversely correlates with protein stability. Proteins with II<40 are predicted as stable. Note the decline in II upon successive exchange of putative phosphorylation sites by phosphomimetic aspartate (D) but not by dephosphomimetic alanine (A) residues.

CENTERFORBIOLOGICALSEQUENCEANALYSIS CBS

NetOGlyc 4.0 Server - prediction results

Technical University of Denmark

```
##gff-version 2
##source-version NetOGlyc 4.0.0.13
##date 13-10-2
##Type Protein
#seqname      source  feature start    end    score    strand   frame    comment
Sequence      netOGlyc-4.0.0.13        CARBOHYD        3        3        0.0237477        .        .
Sequence      netOGlyc-4.0.0.13        CARBOHYD        6        6        0.0140939        .        .
Sequence      netOGlyc-4.0.0.13        CARBOHYD        8        8        0.0154175        .        .
Sequence      netOGlyc-4.0.0.13        CARBOHYD        21       21       0.0422643        .        .
Sequence      netOGlyc-4.0.0.13        CARBOHYD        23       23       0.041661         .        .
Sequence      netOGlyc-4.0.0.13        CARBOHYD        26       26       0.0253381        .        .
Sequence      netOGlyc-4.0.0.13        CARBOHYD        33       33       0.965155         .        .        #POSITIVE
Sequence      netOGlyc-4.0.0.13        CARBOHYD        41       41       0.990921         .        .        #POSITIVE
Sequence      netOGlyc-4.0.0.13        CARBOHYD        56       56       0.989681         .        .        #POSITIVE
Sequence      netOGlyc-4.0.0.13        CARBOHYD        59       59       0.99397 .        .        #POSITIVE
Sequence      netOGlyc-4.0.0.13        CARBOHYD        66       66       0.985554         .        .        #POSITIVE
Sequence      netOGlyc-4.0.0.13        CARBOHYD        70       70       0.952655         .        .        #POSITIVE
Sequence      netOGlyc-4.0.0.13        CARBOHYD        73       73       0.671541         .        .        #POSITIVE
Sequence      netOGlyc-4.0.0.13        CARBOHYD        74       74       0.790337         .        .        #POSITIVE
Sequence      netOGlyc-4.0.0.13        CARBOHYD        77       77       0.615299         .        .        #POSITIVE
Sequence      netOGlyc-4.0.0.13        CARBOHYD        93       93       0.0288631        .        .
Sequence      netOGlyc-4.0.0.13        CARBOHYD        94       94       0.0537127        .        .
Sequence      netOGlyc-4.0.0.13        CARBOHYD        104      104      0.0352132        .        .
Sequence      netOGlyc-4.0.0.13        CARBOHYD        105      105      0.0407821        .        .
Sequence      netOGlyc-4.0.0.13        CARBOHYD        110      110      0.0453348        .        .
Sequence      netOGlyc-4.0.0.13        CARBOHYD        126      126      0.00905412       .        .
Sequence      netOGlyc-4.0.0.13        CARBOHYD        142      142      0.0832123        .        .
Sequence      netOGlyc-4.0.0.13        CARBOHYD        144      144      0.0126749        .        .
Sequence      netOGlyc-4.0.0.13        CARBOHYD        156      156      0.0469291        .        .
```

30-KPSPKPKPVPSPKPKPVQCPPPPRPSVPSPNPRPVTPPRTPGSSGNSCPIDALKLGVCANVLSSLLNIQL-100

30-KPSPKPKPVPSPKPKPVQCPPPPRPSVPSPNPRPVTPPRTPGSSGNSCPIDALKLGVCANVLSSLLNIQL-100

Suppl. figure S6.

Predicted O-glycosylation sites in AZI1.

The full-length AZI1 protein sequence was analysed using the bioinformatic online tool NetOGlyc, according to which "scores higher than 0.5 are predicted as glycosylated and marked with the string "#POSITIVE" in the comment field". The relevant region comprising all predicted sites is displayed at the bottom. Nine candidate target sites for O-glycosylation (blue) are contained in the proline-rich domain of AZI1. Five of these (Ser33, Ser41, Ser59, Thr66, Thr70) overlap with putative MAPK target sites (red) (see also figure 1).

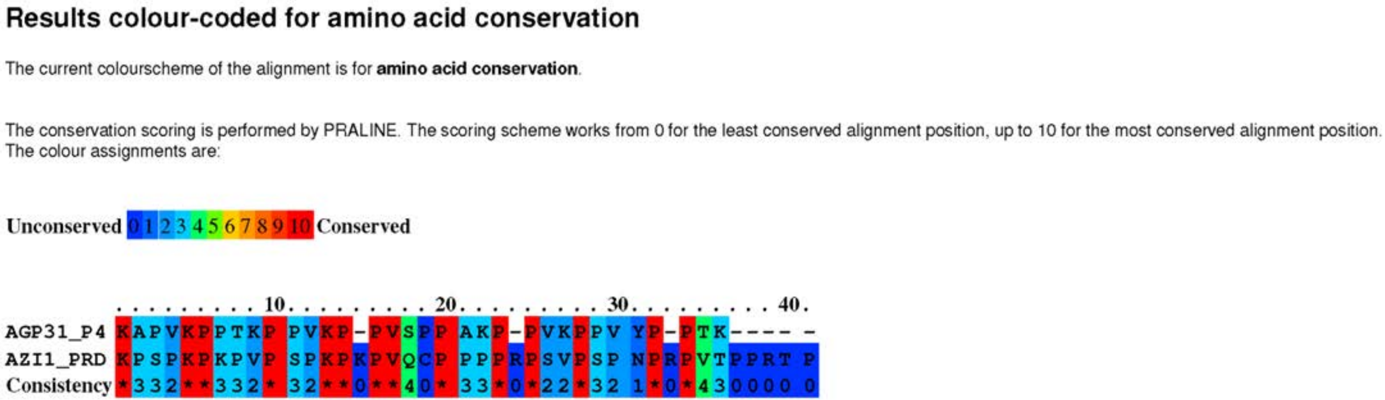

Suppl. figure S7

Alignment of *Arabidopsis* AGP31 sequence of residues 98-129, a reportedly strongly glycosylated peptide (Hijazi et al., 2012), with the proline-rich domain of AZI1. Data were generated using the PRALINE software http://www.ibi.vu.nl/programs/pralinewww/.
